# Building on a Solid Foundation: Adding Relevance and Reproducibility to Neurological Modeling Using Human Pluripotent Stem Cells

**DOI:** 10.3389/fncel.2021.767457

**Published:** 2021-11-18

**Authors:** Erin Knock, Lisa M. Julian

**Affiliations:** ^1^Research and Development, STEMCELL Technologies Inc., Vancouver, BC, Canada; ^2^Department of Biological Sciences, Faculty of Science, Simon Fraser University, Burnaby, BC, Canada

**Keywords:** human pluripotent stem cells, neural stem cells, regenerative medicine, human brain development, disease modeling

## Abstract

The brain is our most complex and least understood organ. Animal models have long been the most versatile tools available to dissect brain form and function; however, the human brain is highly distinct from that of standard model organisms. In addition to existing models, access to human brain cells and tissues is essential to reach new frontiers in our understanding of the human brain and how to intervene therapeutically in the face of disease or injury. In this review, we discuss current and developing culture models of human neural tissue, outlining advantages over animal models and key challenges that remain to be overcome. Our principal focus is on advances in engineering neural cells and tissue constructs from human pluripotent stem cells (PSCs), though primary human cell and slice culture are also discussed. By highlighting studies that combine animal models and human neural cell culture techniques, we endeavor to demonstrate that clever use of these orthogonal model systems produces more reproducible, physiological, and clinically relevant data than either approach alone. We provide examples across a range of topics in neuroscience research including brain development, injury, and cancer, neurodegenerative diseases, and psychiatric conditions. Finally, as testing of PSC-derived neurons for cell replacement therapy progresses, we touch on the advancements that are needed to make this a clinical mainstay.

## Introduction

The brain is the most complex organ in the human body, estimated to contain over 85 billion neurons and a similar number of non-neuronal cells (Azevedo et al., 2009). Specialized neuron subtypes continue to be identified and characterized at an increasing rate (Chen et al., 2017; Zhong et al., 2018; Hodge et al., 2019; Ximerakis et al., 2019; Polepalli et al., 2020; Bakken et al., 2021; Berg et al., 2021). The diverse non-neural populations that populate our brains include astrocytes, oligodendrocytes, neural stem and progenitor cell subtypes, and those with mesenchymal (microglia), epithelial (choroid plexus) and endothelial (vasculature) origins. Cell types of the brain are highly varied molecularly and functionally, displaying unique signatures of electrical activity, neurotransmitter release, inflammatory responses, synaptic pruning and blood-brain barrier mechanisms. Our understanding of these complex features, how they're driven and how their intersection is important for brain function, remains quite limited. The plasticity of neural networks as they respond to and process stimuli is therefore only beginning to be elucidated. Understanding the remarkable synergy by which the vast number of cells in the brain connect together to produce human emotion and behavior is one of the richest and most exciting frontiers of science.

**Figure 1:**
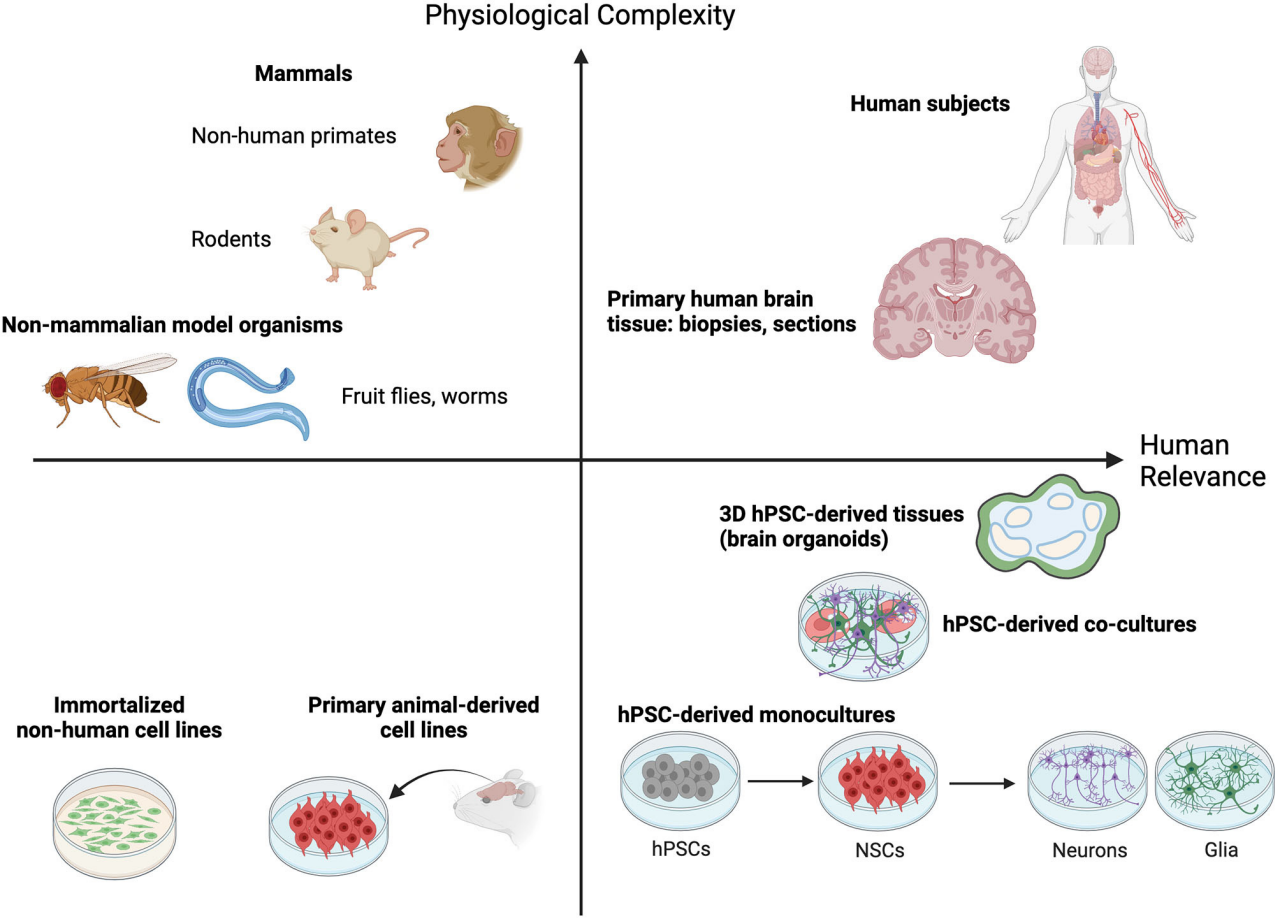
Animal and cell culture models fall on a continuum that balances providing physiological complexity with human brain relevance. Non-mammalian model organisms like worms (*C. elegans*) and fruit flies (*D. melanogaster*) allow molecular and functional investigation of simple neuronal circuits and rational dissection of critical regulatory pathways in a physiologically relevant setting. Rodent and non-human primate models provide additional physiological complexity by permitting quantitative analysis of complex behavioral traits and simultaneously enrich relevance to the human condition. Human subjects and dissected human brain tissues fall high on both measures, but their procurement is rare or ethically impossible. The emergence of human pluripotent stem cell (hPSC)-derived 2D cell and 3D tissue models offer a highly needed source of neurological modeling tools with high relevance to the human brain. Though they offer less physiological complexity than animal models this capacity is ever increasing as technology advances (defined cell co-cultures and 3D tissue engineering). Furthermore, their relevance to the human condition is greatly enhanced compared to traditional culture of immortalized or non-human primary cell lines. A combination of hPSC-based and animal model approaches offers the best opportunity to capture both physiological complexity and relevance to the human brain.

The pursuit of decoding the mysteries of the nervous system has led to a wealth of current tools that are enabling us to investigate increasingly transformative research questions. It goes without saying that human brain tissue is not easy to obtain from the source. While some behavioral studies performed in human subjects have provided insights, performing such experiments in a scientifically rigorous and ethical manner is challenging. Innovative neuroscientists over the decades have found new strategies to explore the answers they seek that balance physiological complexity with increasing relevance to the human brain (Figure 1). The simplest neural circuits can be modeled in fruit flies, zebra fish and roundworms (Guo et al., 2019; El-Daher and Becker, 2020; Singh and Aballay, 2020). Inbred rodents and chick embryos allow for straight-forward genetic manipulation and permit studies to link DNA sequence with consequent effects on cell biology, tissue development, maintenance and function, and organismal behavior (Keynes and Cook, 2018; Gulinello et al., 2019). Primates, like chimpanzees and macaques, can model some aspects of more complex social behaviors and disorders when rodents fall short (Shen, 2016). Traditional animal models used to dissect brain form and function have provided critical insights, informing our current understanding of the basic mechanisms of neurobiology. For example, work done in mice and rabbits was essential to our understanding of how acetaminophen acts as an analgesic (Swierkosz et al., 2002). Our knowledge of neural tube formation and closure was borne from work with frog embryos (for one example see Lutz et al., 1999). The neurotoxic effects of 1-methyl-4-phenyl-1,2,5,6-tetrahydropyridine (MPTP) in creating Parkinson's-like symptoms were not fully resolved until the drug was administered to squirrel monkeys (Langston et al., 1983).

Despite these advances, it's clear that research using human cell sources is critical to overcome significant knowledge gaps that remain about the human brain (Figure 1). This is underscored by the existence of basic foundational differences in brain evolution across species. The fact that rodent cortices are smooth (lissencephalic) while the human cortex is highly folded (gyrencephalic) illustrates the point. Recent single cell RNA-sequencing of the middle temporal gyrus from both mouse and human tissue found that while most cell types are broadly conserved, their relative proportions, laminar distributions, morphologies and gene expression patterns are vastly different between the two species (Hodge et al., 2019). A similar conclusion was drawn from analyses of single cells derived from the primary motor cortex of humans, the non-human primate marmoset, and mice (Bakken et al., 2021). While the broad molecular identities of cell types in this region were found to be conserved, their relative proportions, transcriptional and chromatin states, and expression patterns of cell type marker genes were quite distinct between the three species. Additionally, the mouse brain lacks certain defined structures and cell types that are found in humans. These include a greater number of glutamatergic neuron subtypes in the supragranular region of the human cortex (Hodge et al., 2019; Berg et al., 2021) and the human outer subventricular zone (oSVZ). The oSVZ is populated with basal radial glia cells, a neocortical stem cell type important for neuronal expansion and cortical folding in gyrencephalic species; in line with the expanded oSVZ in humans, basal radial glia are abundant in humans and rare in mice (Pollen et al., 2015). Additionally, at the sub-cellular and physiological levels, several proteins known to be involved in human disease, such as amyloid precursor protein, don't display the same pathological features in mice.

A formidable constraint, the vast complexity of human behaviors cannot be accurately modeled in other species. For example, stress has been shown to be one key indicator of depression in humans. Several types of behavioral tests have been established to measure stress in mice, however it has been difficult to meaningfully link the results of these standardized tests to depression-related coping mechanisms typical of humans (see Commons et al., 2017 for one key example). The consequences of inter-species evolutionary and physiological differences are exemplified by research into many developmental and age-related neurological disorders, including Alzheimer's disease, in which rodents are still the prevailing model. The most telling outcome of this has been the large numbers of successful drugs that reverse characteristic biochemical hallmarks and memory defects in mice which subsequently fail in human clinical trials (Ransohoff, 2018). One report mentions that a single strain of mice used to model Alzheimer's Disease was “cured” over 300 times, though not one drug reported clinical success (Zahs and Ashe, 2010). Thus, many questions related to human specific mechanisms of development and disease for which animal models have yielded insights will inevitably require replication and additional study in relevant human models.

## Culture Models for Neuroscience Research

### Primary Human Tissue

Advances in brain mapping are increasingly providing a better understanding of the firing patterns of neuronal networks within distinct brain regions. With these rich data sets, correlations can be made between human behavior, disease states, and the functional areas of the brain that are affected (Mitchell et al., 2019). Though to achieve the widely sought goal of successful pharmacological intervention, deeper investigation into these processes at the cellular and biochemical levels is needed. While many current efforts in neuroscience research are providing deep knowledge of molecular and functional neurological mechanisms, correlating *in vitro* and *in vivo* observations remains a significant challenge. Human cellular models, especially those that mimic the associated *in vivo* tissue environment, provide the best bridge to form that link.

The most relevant source of *in vivo* neural cells is the human brain itself (Figure 1). On rare occasions, access to living patient tissue can be granted, though most studies rely on donation of post-mortem tissue. When access to tissue is possible, biochemical analysis of organotypic slice culture allows for the characterization of human-specific cell and molecular phenotypes of disease. In a recent example the existing *in vivo* structures present in organotypic slice culture made it possible to examine the spreading of α-synuclein aggregates between neurons (Elfarrash et al., 2019). Having direct access to the living tissue allowed the authors to show that spreading of this pathogenic protein can occur even in the absence of disease characteristic α-synuclein Serine 129 phosphorylation. Neural stem cells have also been isolated from human embryonic brain tissue and can be expanded in culture and differentiated to downstream neural cell types (Dos Reis et al., 2020). In fact, dopaminergic neurons obtained directly from human embryos were some of the first to be used for cell transplantation experiments in Parkinson's Disease patients. For primary human brain-derived cultures time is the enemy, as most neurons begin to die after a few hours in culture and many neuronal cultures cannot be maintained for longer than 14 days (Croft et al., 2019).

### Pluripotent Stem Cells

**Figure 2:**
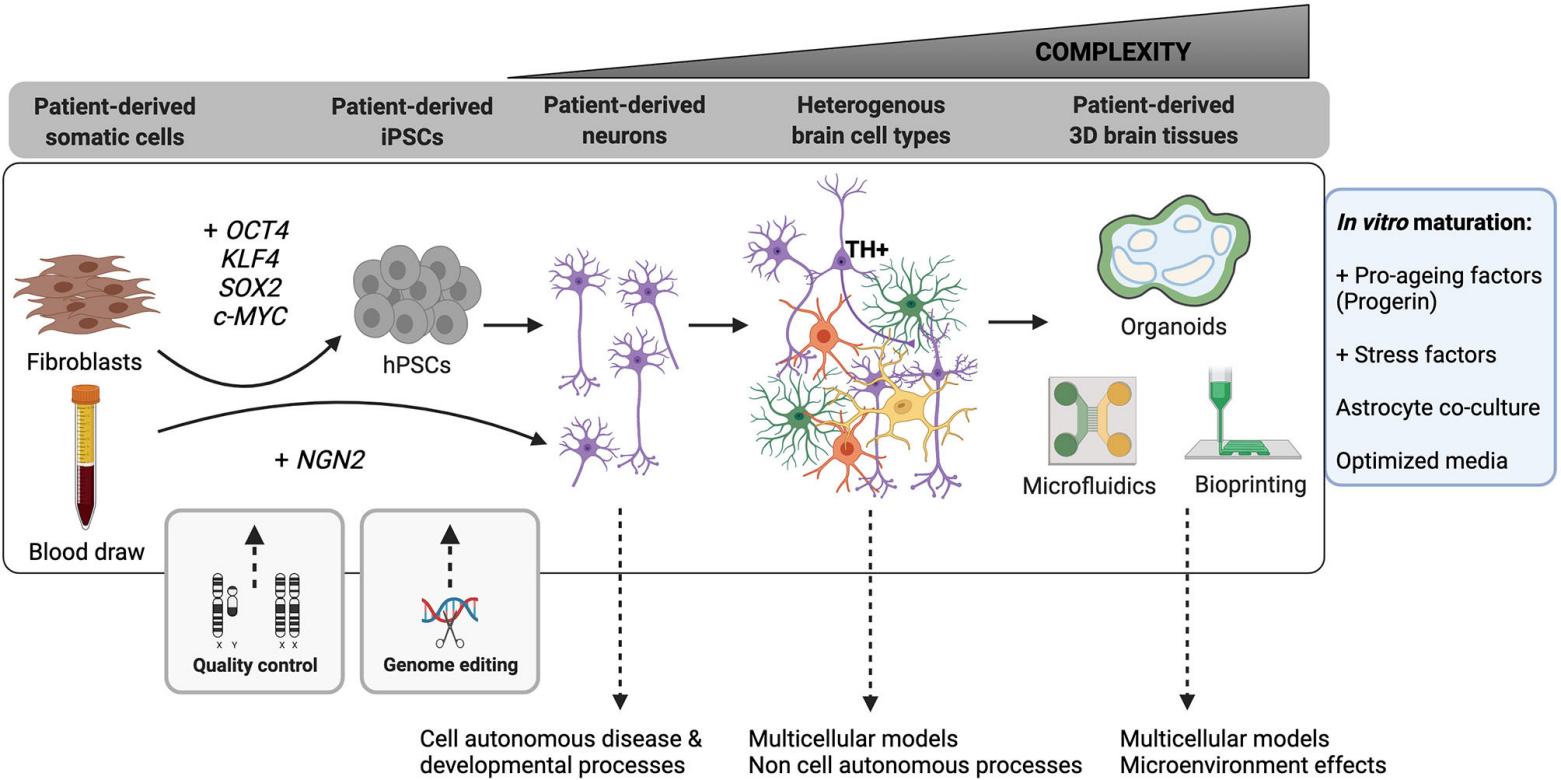
Advances in hPSC-based neurological modeling are increasingly improving the complexity of human neural cell types and circuits we can model. Somatic cells from multiple human patient sources (fibroblast skin cells, blood, urine) can now be used to produce induced human pluripotent stem cells (hPSCs) by over-expression of typically four key pluripotency transcription factors (*OCT4, SOX2, KLF4, c-MYC*). Similarly, induced neurons can be directly established by over-expression of neuronal identity genes like *NGN2*. It is essential to regularly perform quality control assays (detailed in the main text) to ensure maintenance of a high-quality pluripotent state in reprogrammed hPSCs. This stage also offers a critical period for genome editing (e.g., CRISPR-Cas9) in which to introduce mutations of interest or create isogenic control lines. As a field, we can reproducibly generate fetal-stage excitatory and inhibitory neurons in 2D and 3D cell cultures. These cultures permit analysis of cell-autonomous mechanisms of development and disease, especially in the presence of a disease-causing mutation. Recent efforts have increased our ability to generate additional specialized brain-resident cells (“Heterogenous brain cell types”) and to produce more mature neurons (purple = excitatory and inhibitory neurons with elaborate neurite networks; green = astrocytes; orange = oligodendrocytes; yellow = microglia). These heterogeneous cultures permit modeling of non-cell autonomous processes. Offering the most advanced level of complexity in hPSC models are 3D brain tissue engineering approaches, including organoids (rounded enclosed objects = ventricular zones; blue shading = neural stem and progenitor cells; green shading = neurons and other differentiated cell types) and bioprinted constructs, which can incorporate microfluidic channels for advanced microenvironmental control. Studies have also begun to incorporate vasculature and components of the blood-brain barrier into microfluidic and organoid models. The box to the right outlines recent and developing strategies for promoting neuronal maturation and aging in 2D and 3D models.

Pluripotent stem cells (PSCs) are a powerful tool that have allowed us to overcome the limits of tissue availability and low viability of primary human tissue in culture (for a comparison of typical neural culture systems see Table 1). Now researchers with access to readily available somatic cell sources such as blood, skin and urine from normal or disease donors, can establish induced pluripotent stem cell (iPSC) lines to study their disease of interest through the introduction of core pluripotency transcription factors (such as OCT4, SOX2, c-MYC, Lin28, NANOG, and KLF4). These iPSC cultures can then subsequently be directed to differentiate into almost any cell type of interest using well-established protocols, permitting the derivation and analysis of distinct cell types along major lineages (Chambers et al., 2009; Nolbrant et al., 2017; Tcw et al., 2017). Publically available cell banking initiatives, through which researchers can acquire already established iPSC lines for study, are also now widely available [e.g., International Stem Cell Banking Initiative (https://www.iscbi.org/), Coriell Institute for Medical Research (https://www.coriell.org/), European bank for induced pluripotent stem cells (www.ebisc.org), StemBANCC (www.stembancc.org), HipSci (www.hipsci.org), National Institute of Neurological Disorders and Stroke (NINDS) Human Cell and Data Repository (https://stemcells.nindsgenetics.org/), Simon's Foundation (https://www.sfari.org/resource/ips-cells/)]. The flexibility of human PSC culture systems is well-suited to studies aiming to discern cell autonomous vs. non cell autonomous effects by comparing phenotypes in pure cultures vs. heterogeneous co-cultures, lineage tracing across multiple cell types, and genome editing to permit comparison of control and isogenic edited lines (Figure 2).

**Table 1:** Comparison of typical neural culture systems.

**Culture System**	**Application**	**Pros**	**Cons**
Organotypic slices	- Complex neural circuit modeling - Tumor invasion analysis	Retain *in vivo* organization	- Limited culture life - Genetic manipulation difficult
Primary neurospheres	- Neural stem and progenitor cell expansion - Tumor cell expansion	- Isolation of mature (active) cell types and pure cell populations possible - Co-culture of distinct cell types possible - Low variability between experiments - Ease of expansion for high cell yield	- Limited passage number - Technically difficult to passage - Need sufficient starting cell source - Difficult to establish from adult tissues - Lacks *in vivo* organization and diverse cell types
Primary cell adherent culture	- Separating cell autonomous from non-autonomous effects	- Isolation of mature (active) cell types and pure populations of some cell types - Co-culture of distinct cell types possible - Low variability between experiments	- Isolating pure cell types difficult and time consuming - Difficult to establish from adult tissues - Genetic manipulation difficult outside of proliferating NPCs - Limited culture life-span for mature cell types - Lacks *in vivo* organization and diverse cell types
hPSC-derived adherent culture	- Separating cell autonomous from non-autonomous effects - Human genetic studies (background variability and single gene mutations) - Neurogenesis and gliogenesis	- Accessible human relevant cells - Pure culture and co-cultures possible - Can generate specific cell subtypes - Easy to produce multiple cell types from one initial source - Early proliferating cell types (PSCs or NPCs) are expandable and bankable - Genetic manipulation protocols well established	- Cells produced are typically fetal-like - Lacks *in vivo* organization - Long differentiation protocols and maturation times - Mastery of hPSC culture a prerequisite - High cell line to cell line variability
hPSC-derived spheroids and organoids	Tumor cell expansion, Modeling brain development and neurodevelopmental disorders	- High efficiency of neural differentiation - Observe interactions between multiple cell types *- In vivo*-like organization - Spontaneous (unpatterned) or directed (patterned) differentiation possible	- Similar to adherent culture - Lacking some critical non-CNS cell types (e.g., microglia and vasculature), though these can be added by co-culture

In addition to iPSC reprogramming and subsequent directed lineage differentiation, transdifferentiation using cell-type specific transcription factors allows direct reprogramming of one somatic cell type into another (Figure 2; for example, fibroblasts to neurons: Vierbuchen et al., 2010). This approach has the advantage of maintaining the epigenetic signature of the cell type of origin by avoiding transition through an embryonic iPSC state, which is especially useful when modeling diseases of aging (Huh et al., 2016). Similarly, forward programming of PSCs using transcription factor overexpression can produce highly pure populations of neurons (Zhang et al., 2013), astrocytes (Li X. et al., 2018) and oligodendrocytes (García-León et al., 2018). The speed and ease of use of these protocols has made them especially attractive for large scale screening applications (Sridharan et al., 2019). The most direct clinical application of somatic cell reprogramming is the potential for *in vivo* transdifferentiation of resident astrocytes into functional neurons for neuronal cell replacement therapy (Liu et al., 2015; Aravantinou-Fatorou and Thomaidou, 2020; Ma et al., 2021). This strategy, which has been achieved with both neuronal transcription factor expression and more recently targeted small molecules, could importantly circumvent the need to generate large numbers of cells for transplant and the worry of immunocompatibility. Questions remain, however, about the true identity, functionality, and origin of the resulting cell types if developmental programs are not strictly followed and when artificially high levels of cellular factors are expressed (Nehme et al., 2018; Wang et al., 2021). In fact, a recent group pointed out that neurons that were presumed to be reprogrammed from astrocytes were actually, in fact, derived from other endogenous neurons (Wang et al., 2021). This highlights the need for a closer look at the consequences of somatic cell reprogramming, including a necessity to incorporate careful lineage tracing analyses to ensure we understand which cell identity transitions are in fact taking place.

A more recent advance in stem cell research has been the development of differentiation protocols in three-dimensional cultures (Figure 2; see Tian et al., 2020 for a recent review), including both spheroids (aggregates of differentiated cells) and organoids (structures that form out of aggregated progenitors). The first neuroectoderm-derived organoids (Eiraku et al., 2011) were able to recapitulate many features of the developing eye. Later protocols exploited the intrinsic self-organizing potential of aggregated neural precursors to activate endogenous cell signaling mechanisms to promote spontaneous differentiation into cerebral cortex tissue, mimicking similar developmental stages and tissue architecture as the developing human brain (Lancaster et al., 2013). Similar to 2D differentiation protocols, cytokines and small molecules can be added to pattern the organoids into specific cortical regions (Muguruma et al., 2015; Jo et al., 2016; Birey et al., 2017; Sloan et al., 2018; Marton et al., 2019; Eura et al., 2020; Hor and Ng, 2020). The primary advantage of organoid culture technology is the ability to replicate cell organization and maturation during development while simultaneously maintaining cell viability in long term culture (see Table 1). Using these sophisticated human tissue models, researchers have also been able to fuse organoids that represent adjacent anatomical regions. This permits modeling of the connections between neurons from different areas of the brain (Bagley et al., 2017) and between the eye and brain (Fligor et al., 2018). These same principles have also been applied to cancer research, such that organoids derived from patient biopsies more closely mimic the attributes of the parent tumor than do traditional immortalized cancer cell line models (Hubert et al., 2016; Jacob et al., 2020).

### Considerations for Starting PSC Cultures

Starting a project using PSCs for the first time is not without a learning curve (see Table 2 and Ernst, 2020 for an in-depth review). However, advances in the field have lowered many barriers to adoption. Robust reagents and methods exist for embryonic stem (ES) and iPSC culture, as well as neural differentiation. While it may never be possible to fully recapitulate every neural subtype that exists in both space and time, protocols have been established to generate most major cell types (Chambers et al., 2009; Falk et al., 2012; Douvaras et al., 2014; Tcw et al., 2017; Marton et al., 2019). Addition of protein and small molecule patterning factors specifies regionality to these protocols to generate even more specialized cell types like dopaminergic (Kirkeby et al., 2012), hippocampal (Sarkar et al., 2018), serotonergic (Lu et al., 2016), and motor neurons (Hu and Zhang, 2009). Protocols for neural crest cell differentiation (Hackland et al., 2017; Tchieu et al., 2017) enable the generation of peripheral neurons (Prince et al., 1991) as well as their mesenchymal derivatives (cartilage, bone (Leung et al., 2016), fat (Gomez et al., 2019) and smooth muscle (Serrano et al., 2019; Delaney et al., 2020; Li X. et al., 2020). Even non-ectodermal contributors to the nervous system can be produced and incorporated into brain tissue models including microglia (Abud et al., 2017; McQuade et al., 2018), brain microvascular endothelial cells (Qian et al., 2017) and pericytes (Stebbins et al., 2019).

**Table 2:** Considerations for PSC culture.

**Source material**	**Study design**	**Reprogramming method**	**Replicates**
**In-house derived hPSCs:** - Dependent on access to somatic cell sources (skin, blood, urine), ease and efficiency of reprogramming (e.g., urine is easily accessible, but more laborious than other sources to reprogram) - Age (younger cells reprogram more efficiently) - Access to patients with mutations of interest can be challenging **Purchased cell lines** (e.g., Coriell, EBISC, StemBANCC, HipSci, NINDS): - Dependent on price, availability of desired mutation, patient medical data - Assess restrictions on use (expansion, banking, differentiation, commercial use) - Verify appropriate patient consent and institutional review forms available (ICF and IRB review and approval)	- Biological replicates are critical: collect from multiple donors where possible - Non affected familial control or isogenic wild-type lines are necessary - For case-control study designs: multiple clones required - Consider genetic engineering if controls or lines with mutation of interest are unavailable	**Integrating vs. non-integrating reprogramming**: - Genomic integration of vector can lead to unwanted secondary mutations and reactivation of reprogramming factors - Certain vectors are more effective with certain cell types (Okita et al., 2013) - Labor cost: non-integrating methods often require specialized equipment (e.g., electroporator) - Financial cost: increasing efficiency typically comes with higher cost - Selection of reprogramming factors (e.g., OCT4, SOX2, c-MYC, KLF4) - Consider published protocols to increase reprogramming efficiency (Huangfu et al., 2008; Esteban et al., 2010) - Selection of high efficiency reprogramming medium	**Accounting for inherent variability among hPSC lines:** - Include hPSCs from at least 3 similarly affected subjects. If not possible, use at least 3 hPSC clones per donor - Ensure all experimental lines are of a similar age - Sex balance: use an equal number of female and male hPSC lines - Consider other potential mitigating health factors from sample (other health issues, diet, potential genetic modifiers)
**Culture Considerations**	**Quality Control**	**Maintenance**	**Differentiation/Maturation Method**
- Dedicated Biological Safety Cabinets and safety protocols for human tissue work - Separate space for human vs. other mammalian culture preferable - Asses incoming human samples for presence of infectious viruses - Mycoplasma testing of all new cell lines - Cryogenic storage essential for biobanking viable hPSCs	- Daily morphological assessment: visibly differentiated cells can be manually removed - Karyotype all new lines, genetic analysis (QPCR for common abnormalities) for new thaws and before important experiments - Assess pluripotency (teratoma or trilineage differentiation) of all new lines - Assess markers of pluripotent state (e.g., OCT4, TRA-1–60) for all new lines	- Choose between defined (feeder and serum free) or undefined (feeders and serum/serum replacement, sometimes more efficient for differentiation) culture conditions - Clump vs. single cell passaging (single cell passaging can increase chromosomal abnormalities) - Matrix: defined (vitronectin, laminin) or undefined (matrigel) - Low protein, robust or stabilized growth media - Regulatory compliance of hPSC growth media- Feed, passaging and banking schedule	- Developmental patterning to drive lineage induction vs. forward programming (e.g., NGN2 overexpression) - Pure populations or co-cultures - 2D (largely homogenous cell types) vs. 3D (e.g., heterogeneous organoids) architecture - Transdifferentiation (retain epigenetic signature of source by skipping PSC stage) - Identify best time-points to freeze and biobank cells, with high post-thaw viability - Maturation strategy (e.g., increased culture time, physiological maturation medium, addition of cell stressors)

Standardized media and protocols have made it easier than ever for labs that have traditionally focused on animal work to adopt hPSC-based culture into their pipelines. Though despite the advances mentioned above, there are limits to the current applicability of stem cell-derived neural cells. Many labs new to stem cell culture, even when experienced in traditional primary or immortalized cell culture, struggle to maintain quality human stem cell lines. This is especially acute when the project involves generating novel iPSC lines from multiple patients. The choice of starting cell type, reprogramming method, clone selection, culture medium and passaging protocol can all influence the efficiency of downstream differentiation (see Table 2 and Ernst, 2020). Knowing how crucial starting cell quality is for downstream differentiation, several large consortia have agreed on a minimum set of criteria required for assessing stem cell quality (Sullivan et al., 2018). Several can be routinely monitored in the lab without specialized training or equipment (morphology, marker expression, trilineage differentiation potential, epigenetic signature). Tracking and maintaining genetic stability is more difficult. qPCR kits exist that will give basic information on common small chromosomal amplifications and deletions known to exist in pluripotent cells (for review see Halliwell et al., 2020). This needs to be combined, however, with G-banding and possible copy number variation analysis to truly understand genetic stability. These later two methods are costly and are therefore usually only done when starting a new cell bank or before a critical experiment. Several publications suggest that even small chromosomal gains or losses influence downstream differentiation efficiency (reviewed in Henry et al., 2018). Labs ignore karyotyping at their peril. For example, gain of chromosome 20 frequently occurs in PSC culture, driven by *BCL2L1*, a gene associated with reduced apoptosis and cancer (Enver et al., 2005; Baker et al., 2007). Cells with amplifications like this have a growth advantage, but with a potential increase in tumorigenic potential (Blum and Benvenisty, 2008; Ben-David and Benvenisty, 2011).

### Addressing Long Time Frames for *in vivo* Maturation

An important consideration is the immature fetal-like nature of PSC-derived neural cell types. Several approaches are being taken to increase the speed at which neurons are produced and become functionally mature *in vitro*. Applying compounds, such as inducers of reactive oxygen species, to induce age-associated cellular stressors has been effective in revealing phenotypes associated with progressive neurodegenerative disease (Mertens et al., 2018) genetic over-expression of pro-aging factors (i.e., progerin) have also been applied and result in increased molecular signatures of aging (Miller et al., 2013; Cornacchia and Studer, 2017). However, to date no single approach has been conclusively proven to generate adult-like functionally mature neurons.

Modifying neural cell culture protocols to increase maturation *in vitro* has, however, yielded some degree of success. Co-culture of neurons with astrocytes has long been shown to increase neurite outgrowth and activity, and this approach is now yielding more physiologically relevant iPSC-derived neuronal cell disease models (Odawara et al., 2014; Kuijlaars et al., 2016; di Domenico et al., 2019; Enright et al., 2020; Leventoux et al., 2020). Cell culture media formulated to mimic human cerebral spinal fluid (Bardy et al., 2015) increases neuron maturation and generates neurons which become active at a much earlier stage in culture. Organoids appear to provide the necessary tissue structure and cell non-autonomous support needed for increased neuronal cell maturation, demonstrating complex oscillatory firing patterns in neurons derived from cerebral organoids (Trujillo et al., 2019; Fair et al., 2020; Giandomenico et al., 2021). However, there is still some debate as to what molecular or activity readouts define a “mature” neuron. These important phenotypic parameters first need to be defined before the field can move forward with improving neuron maturation in culture (Livesey et al., 2016; Ghanavatinejad et al., 2019).

### Dealing With Genetic and Technical Variability

Many recent efforts have focused on identifying and addressing key technical issues to increase the biological relevance and decrease the variability of PSC-derived models (see Volpato and Webber, 2020 for a recent review). Genetic variability between cell lines can not only mask phenotypes but also influence differentiation efficiency (Ortmann and Vallier, 2017). One interesting method to overcome this involves “normalizing” signaling pathways by simultaneous activation and inhibition during the first phases of differentiation (Hackland et al., 2017). Other groups have reported that ES/ iPSC with naïve gene expression profiles are better able to differentiate into neural lineages (Park et al., 2018; Watanabe et al., 2020). Further, there is some evidence to suggest that addition of something as simple as low concentrations of DMSO can increase differentiation to neural lineages in difficult to differentiate PSC lines (Li J. et al., 2018); this has been attributed to activation of the Retinoblastoma signaling pathway. While standardized culture media and protocols can overcome much inter-cell line variability, there needs to be a deeper understanding of the signaling pathways involved during the earliest cell fate decisions that take place during differentiation. Normalizing these pathways before differentiation is induced could help increase reproducibility in differentiation efficiency across cell lines, especially PSC lines that are generated using slightly different methods and by different researchers. Increased access to and decreased pricing for single cell genomic studies is continually improving our understanding of variation among human neuronal cells and tissues, and is providing insight into the genetic vs. technical basis of developmental and disease phenotypes observed *in vitro* (van den Hurk and Bardy, 2019).

Traditionally, the main advantage of using animal models has been the ease of genetic manipulation and control over the genetic background. Due to the extensive genetic variability that exists between humans, stem cell-based studies aiming to determine the neurological impacts of mutations in even a single gene have traditionally required a large number of cell lines derived from patients with the same disease (and with multiple clones per patient), as well as multiple cell lines from unaffected individuals to serve as controls. Though more recently, for diseases caused by known genetic variants or with established genotype-phenotype correlations, genome editing technologies such as CRISPR/Cas9 (reviewed in Rehbach et al., 2020) can efficiently establish paired gene variant and isogenic control cell lines. Thus, inbred animals are no longer the only way to model disease-related mutations across isogenic backgrounds. The relative ease by which isogenic control lines can now be engineered in hPSC also greatly increases the opportunity for stem cell-derived models to yield significant phenotypic changes, by limiting genomic variability among experimental samples without the need to establish and analyze a cost-prohibitive number of patient and control cell lines.

For diseases with complex genetics or combined environmental and genetic contributions, human cell-based studies still need to rely on genetically related or cohort matched unaffected controls (see Riemens et al., 2020 for a recent review). In some cases, it can also be argued that to develop effective disease treatments study designs need to reflect the inherent genetic variability in the population they are aiming to treat. In animal models this is accomplished by testing different strains and stratifying study subjects by sex, age, diet, and environment. With stem cell-derived models this same level of control can be accomplished by incorporating samples from multiple patients, deriving multiple clones from each line, creating multiple sets of isogenic variant and control lines (in the case of single gene mutations), and adopting a standardized cell culture protocol with carefully implemented quality control measures while maintaining diverse hPSC lines. When modeling phenotypes that underlie development or disease states, assessing variability between cell lines established from different donors is particularly critical; increasing access to and streamlined strategies to analyze iPSC lines is continually making these endeavors more possible (Schuster et al., 2015). An effective way to establish the validity of an observation in the face of high biological variability is to test it across multiple experimental models and methods. In one study (Yuva-Aydemir et al., 2019), the authors modeled Amyloid Lateral Sclerosis (ALS) by producing Drosophila mutants with expanded GGGGCC repeats in *C9ORF72*. With this approach, axonal and locomotor defects that reflect those observed in ALS patients were reproduced. The simplicity of Drosophila genetics allowed them to perform an unbiased genetic screen in which they discovered that partial loss of Lilliputian activity can suppress the toxicity of the repeat expansion. Using CRISPR-Cas9 genome editing they were then able to knockout one of the mammalian homologs of Lilliputian in iPSC derived from ALS patients with *C9ORF72* repeat expansions, in which they observed a decrease in TDP-43 pathology and axonal degeneration. Subsequently, Anastasaki et al. used CRISPR-Cas9 to engineer seven pairs of isogenic iPSC lines covering a range of mutations in *NF1*, the causative gene in the developmental disorder Neurofibromatosis Type 1 (Anastasaki et al., 2020). As expected, some phenotypes were common across all cell lines (increased proliferation, RAS pathway activity) while some varied according to the particular mutation (dopamine levels, apoptosis and neuron differentiation). In order to determine which phenotypes were likely to be disease related the authors compared their results to those collected in *Nf1*-mutant mice (which also represented a range of mutations). Notably they were able to replicate the finding that dopamine levels are reduced as a result of some, but not all, *NF1*/*Nf1* mutants in mice/humans.

Both of these studies demonstrate how replication of phenotypes across model systems increases the confidence in disease-associated observations. Pinpointing phenotypes that persist across such wide variability in genetic background will help to identify treatment targets with the most wide-spread effect. In the next section we will discuss disease specific examples of this strategy.

## Complementing and Building on Animal Models With Human Stem Cell Models

Uncovering disease-causing mechanisms and effective therapeutic strategies are expensive, high stakes undertakings. Collecting multiple data sets from orthogonal model systems to support these efforts greatly enhances the likelihood of success. Below are some recent examples where combining human and animal models of disease have resulted in new discoveries of disease mechanisms and promising treatments.

### Neurodegeneration

Neurodegenerative diseases, particularly those with a protein aggregation component, have been especially hard to model accurately in animals like mice. As such, PSCs have become a very attractive model for this field (for more detailed discussions on the topic see: Riemens et al., 2020; Valadez-Barba et al., 2020; D'Souza et al., 2021). Alzheimer's disease, Parkinson's disease, ALS and Huntington's disease are complex disorders with the vast majority being sporadic, but with familial cases caused by known mutations that provide evidence of strong genetic risk factors, such as ApoE4 (Corder et al., 1993). Neuronal cell death is a hallmark of most of these disorders, making cell replacement therapy an attractive treatment option. Replacement of dopaminergic neurons for treatment of Parkinson's disease has been advancing rapidly with notable recent success (Duma et al., 2019; Takahashi, 2020; Piao et al., 2021). Stem cell-based models have been used to recapitulate most, if not all, disease phenotypes including immune and vascular pathologies (Blanchard et al., 2020; Liu et al., 2020; McQuade et al., 2020; Zhao J. et al., 2020). Organoids are particularly attractive models as some protein aggregate pathologies are only observed in 3D culture systems (see Venkataraman et al., 2020; Cenini et al., 2021 for recent reviews). Incredibly, this is despite the fact that PSC-derived neurons are fetal in nature; though this may be helpful in detecting pre-clinical abnormalities (Malankhanova et al., 2020). Still, age is the primary indicator for the majority of these conditions. Although approaches such as transdifferentiation (see Mertens et al., 2021 for a recent example) to generate “aged” neurons are being developed, combining animal models and pluripotent stem cell technology is especially useful in modeling the breadth of phenotypes in these diseases.

An approach taken by Höing et al. (2012) combined mouse ES cell-derived motor neurons and astrocytes with a transformed human microglia cell line (BV2). This co-culture model was developed as a cost-efficient way to scale up for a high-throughput screen. The microglia were activated to mimic a pro-inflammatory environment, which the authors hypothesized was a contributing factor in sporadic ALS. The activated microglia induced cell death in the neighboring motor neurons and the authors screened for compounds that could reduce this death. One compound they found to be effective, termed E4, was then tested on human PSC-derived neurons. E4 was found to increase neuron survival in the presence of DeltaNO (a known inducer of cell death), with no adverse effects in the absence of DeltaNO. This co-culture approach of mouse and human cell types provided a quick and convenient way to screen compounds in a high-throughput manner, and to assess any potential cell non-autonomous mechanisms of action.

An alternative approach is to identify phenotypes in human cells, and subsequently validate and expand on these findings in *in vivo* animal models. Laperle et al. (2020) derived iPSCs from three individuals with young onset Parkinson's disease with no known family history or predisposing mutations. Dopaminergic neurons derived from those cells accumulated α-synuclein, which was found to be due to an impairment in the lysosomal degradation pathway. Deriving an additional seven control and nine patient lines confirmed this novel phenotype in 8/9 patient lines. Three lysosomal agonists were added to the cells in an attempt to decrease α-synuclein levels. One of those agonists, PEP005, was able to reduce α-synuclein and increase the median TH intensity in human dopaminergic neurons. The activity of PEP005 was subsequently confirmed by stereotactic injection into the striatum of wild type mice, in which a similar decrease in α-synuclein levels was observed. The combination of *in vitro* human and *in vivo* observations have increased the confidence in PEP005 as a potential therapeutic.

In Alzheimer's Disease models, altered mitochondrial morphology and function which manifest as increased AMPK activity and decreased mitophagy, were recently observed using electron microscopy (Fang et al., 2019). To investigate this compelling phenotype further, iPSCs from one sporadic and one familial Alzheimer's patient with a matched control were derived. A decrease in mitophagy was further confirmed in cortical neurons differentiated from these cells as indicated by decreases in several mitophagy related proteins. One hypothesis posited is that neuron loss in Alzheimer's disease could be caused by accumulation of damaged mitochondria, leading to energy deprivation and inflammation. To test if activation of mitophagy could affect Alzheimer's disease pathogenesis in a simple *in vivo* model, *C. elegans* was used to screen for inducers of mitophagy. The screen identified two potential candidate compounds, urolithin A and actinonin. These candidates were able to improve memory defects and reduce Aβ levels in a transgenic *C. elegans* Alzheimer's disease model. The mechanisms behind the action of these drugs were conserved in multiple species' models, as similar effects were seen in an Alzheimer's mouse model. Further investigation of these mice revealed that decreased mitophagy in microglia resulted in an inability to remove accumulated Aβ plaques and an increase in the release of pro-inflammatory cytokines. The conservation of these mechanisms across species suggests that enhancers of mitophagy are a good target for new Alzheimer's disease drugs and that physiological assessment of their effects can be adequately performed in animal models.

### Neurodevelopment

Stem cell-derived neural cells are especially powerful models of embryonic development since differentiation protocols are typically designed to closely mimic key signaling pathways during early embryogenesis (for recent reviews see Imaizumi and Okano, 2021; Sabitha et al., 2021). Stem cell-derived neurons, whether in 2D or 3D culture, have been found to exhibit gene expression signatures that are most similar to early to mid-gestational stages (Handel et al., 2016; Logan et al., 2020). Recently, organoids have been shown to display electrical firing patterns that are similar to fetal signals detected by electroencephalography, providing a potential connection between *in vitro* models and *in vivo* phenotypes (Trujillo et al., 2019; Fair et al., 2020; Matsui et al., 2020; Giandomenico et al., 2021). This has led to discoveries using organoids to model fetal toxin exposure (Prince et al., 2019; Arzua et al., 2020) and developmental disorders (Gomes et al., 2020). In a novel application of the technology, organoids are being generated from across several species, leading to discoveries about how brain development has changed over evolution (Kanton et al., 2019; Benito-Kwiecinski et al., 2021; Chan et al., 2021). Additionally, protocols have recently been developed to generate human neural crest cells and their derivatives. A transient population in the embryonic ectoderm, neural crest cells are difficult to isolate and study even in animals. Neural crest cell culture allows for the study of neurocristopathies (Barrell et al., 2019) and neural tube development (Rifes et al., 2020; Libby et al., 2021). These features make stem cell-derived ectoderm cell types including neurons ideal tools for studying neurodevelopmental disorders, and mechanisms of normal brain development and evolution across species.

The Zika virus epidemic of 2015-2016 yielded important advances in modeling vulnerabilities of neurodevelopment using hPSC-derived models (Cavalcante et al., 2020; Pettke et al., 2020; Krenn et al., 2021). Gabriel et al. (2017) took advantage of the ability of cerebral organoids to model microcephaly, a main phenotype in infants born to ZIKA infected mothers. Using two novel and one established strain of Zika virus, the authors found productive infection in 2D cultures of neural progenitor cells (NPCs). While all three strains decreased NPC proliferation one of the novel strains had a far more pronounced effect, showing less spontaneous neuron differentiation and increased apoptosis compared to the other strains. The mechanism of NPC disruption was found to be linked to centriole disruption, with abnormal recruitment of Cep152, PCNT, Cep164, and CPAP proteins. Using infected cerebral organoids the authors linked the centriole disruption to the decrease in proliferation by showing altered planes of division of NPCs in the putative “ventricular zones” of the organoids. As occurs *in vivo*, altered planes of division influence whether NPCs divide symmetrically to self-renew or asymmetrically to generate neurons. The Zika infected NPCs in the organoids skewed toward asymmetric divisions, increasing the number of neurons but consequently depleting the NPC pool. As the organoids continued to develop in culture the lack of NPCs resulted in fewer overall neurons being produced and smaller organoids, thereby replicating the decreased cortical size characteristic of microcephaly. By combining viral infection and organoid technologies the authors were able to correlate the severity of the strain with mechanism of action in driving aberrant brain development: either an increase in apoptosis or an increase in premature neuronal differentiation.

The previous example illustrates how a combination of homogenous populations of 2D cells with more complex organoid models can help reveal *in vivo* mechanisms of disease. However, while organoids can replicate some important features of human brain development like cortical folding (Li et al., 2017; Karzbrun et al., 2018) and abundant outer radial glia (Bershteyn et al., 2017; Andrews et al., 2020; Eze et al., 2021), complex interconnections, neuron remodeling, and synaptic pruning are still best modeled *in vivo*. Toward this goal, Real et al. (2018) injected human iPSC-derived neurons into the cortices of young mice and followed their integration and maturation at a single-cell level over time using *in vivo* imaging. Real-time tracking revealed that more neurons extend processes than in *ex vivo* culture and that those extensions occur more quickly than retraction of processes. This resulted in a pattern of neurite gains and losses that changed dynamics the older the graft was. When neurons derived from Down Syndrome patients were stereotactically injected into adult mouse brains, fewer extensions and retractions were observed, with an increase in neurite stability and reduced activity. This combination of approaches demonstrates an interesting developmental paradigm for human neurons and a new mechanism for Down Syndrome pathology at the synapse level.

### Psychiatric Disease

The most difficult brain pathologies to model are those with a primary behavioral manifestation, as so many are unique to humans as a species. In these cases, correlating molecular and cellular phenotypes from human cells to specific behaviors in animal models provides our strongest evidence for promising therapeutic targets. When using PSC-based models this connection can be made by linking electrophysiological function of PSC-derived neurons to EEG readings from human patients (see (Wang M. et al., 2020)) for a review and (Naujock et al., 2020) for a recent example). Mechanistic and safety studies for new psychiatric drugs can also be worked out in advance of or aligned with clinical trials (see Marcatili et al., 2020 for a recent example using ketamine as an antidepressant). A recent advance using co-cultured organoids patterned to different brain regions (AssemBloids™) opens the possibility to study connectivity issues between specific areas of the brain (reviewed in Marton and Paşca, 2020). This is of particular interest to psychiatric research as pathology often involves signaling between multiple brain regions. The genetics of psychiatric disease are also very complex (Prytkova and Brennand, 2017).

One group (Sawada et al., 2020) used pairs of discordant twins with psychosis to examine the developmental origins of the disease. Single cell RNA sequencing in cerebral organoids from the affected and unaffected twin revealed that organoids from the affected twin had a decrease in the proportion of SOX2+ progenitor regions with a concordantly higher proportion of GABAergic inhibitory type neurons. This phenotype was reversed when the organoids were treated with LiCl, a GSK3β inhibitor and antipsychotic. This suggested an underlying imbalance in the excitatory/inhibitory network of neurons early in embryogenesis as a mechanism for the development of psychosis. This striking result was confirmed in organoids produced from three additional pairs of discordant twins. A new technique aimed at combating the high genetic variability between patients is the so-called “population in a dish,” where many bar-coded PSC lines are combined in a single dish and interrogated together. Single-cell RNA seq can then be used to create phenotypic “sub groups” for further analysis (see Cederquist et al., 2020 for an example).

Shao et al. took a different approach by generating iPSC lines from 14 unaffected and 14 Schizophrenia patients (Shao et al., 2019). RNA-seq performed on 8 week old neurons derived from these iPSC lines indicated that neurons from all individuals had very similar gene expression patterns. Only a few genes, including *PCDHA2* and other protocadherin family members previously linked to Schizophrenia, were differentially expressed between affected and unaffected lines. Examination of a *Pcdha* KO mouse model found decreases in VGAT+ staining on parvalbumin positive neurons with a decrease in neurite length and branch number, suggesting a defect in inhibitory neuron development and function. When the control and patient derived neurons were transplanted into 5–7 week old mice via stereotaxic injection, a mild but significant defect in inhibitory synapse formation was observed. This study suggests a novel link between protocadherin gene expression and inhibitory synapse formation.

Together these studies, using 2D and 3D culture of hPSC-derived neural cells in conjunction with animal models, raise interesting questions about the balance that inhibitory and excitatory neurons play in psychiatric disease. These efforts suggest that even more complex physiological models can be developed using stem cell derived 3D cultures.

### Brain Injury

Repair and regeneration of damaged neurons has been a longstanding goal for regenerative medicine (see Farzaneh et al., 2020 for a recent review). In addition to transplanted mesenchymal stromal cells (MSCs), neural progenitors have also been the subject of testing for clinical trials (eight on-going or completed www.clinicaltrials.gov) for brain repair (Nieves et al., 2020) or spinal cord injury (Kamata et al., 2021). Recognizing the key role they play in CNS function, glia are being considered for replacement as well (Balakrishnan et al., 2020). This is particularly relevant to the treatment of myelination disorders like multiple sclerosis (Smith et al., 2021). An emerging field that has developed tangentially to this is the development of biomaterials to increase PSC-derived graft survival and efficacy (reviewed in Lacalle-Aurioles et al., 2020). An interesting alternate approach is the *in vivo* transdifferentiation of glia/glial progenitors into functional neurons or oligodendrocytes (reviewed in Qian et al., 2021). This approach side-steps the need for host immune matching and graft survival, however more work is needed to fully understand the resulting cell type, it's function and connectivity. Organoids are also being considered as tissue sources for transplantation, though primarily for retinal replacement (Lin et al., 2020), due to the increased maturation of cells in 3D structures. Additionally, organoids are proving a robust injury model, whether it be the consequences of reduced oxygen to the brain after systemic injury (Kim M. S. et al., 2021) or direct traumatic injury to the brain tissue itself (Shi et al., 2021).

Testing PSC-derived cells for cell replacement necessitates safety and efficacy studies *in vivo*, which benefits from even simpler models like Zebrafish (Tayanloo-Beik et al., 2021). To support those studies, Shiga et al. looked at ways to improve autologous transplantation of iPSC-derived neural progenitors (Shiga et al., 2019). The authors tested a previously identified activator of fibrinolysis, an enzymatically inactive tissue-type plasminogen activator (EI-tPA), known to promote neuron growth on iPSC-derived NPCs. EI-tPA increased expression of neural progenitor markers and reduced thrombin-mediated apoptosis *in vitro*. The NPCs were then implanted into a rat model of spinal cord injury either with or without pre-treatment with EI-tPA. The EI-tPA treated NPCs significantly increased motor recovery, body weight and tibialis weight over rats injected with untreated NPCs. The EI-tPA treated NPCs also showed increased survival in the graft (16 weeks) and generated neurons which projected into the ventral spinal T7 segment of the spinal cord with no negative effects on sensory function.

Another group recently built on what was known about the promising effects of MSCs in transplant grafts (Zheng et al., 2021). MSCs don't generate new neural cells in grafts meant to repair brain injuries, but instead secrete extracellular vesicles (EVs) that contain substances which promote cell growth and regeneration. The authors asked whether EVs secreted from NPCs had similar effects. NPCs and MSCs were isolated from newborn mouse subventricular zones and adipose tissue, respectively, in order to collect enough material for the study. EVs were isolated from these cells using two common methods, PEG and ultracentrifugation, and the cargo they contained was characterized. Mass spectrometry revealed the expected cytosolic proteins, transmembrane proteins and EV markers common to most EVs. A selection of miRNAs with known neuroprotective function was also detected by qPCR. To determine their potential protective properties the EVs were added to the culture medium of iPSC-derived cerebral organoids exposed to oxygen-glucose deprivation as a stroke model. The EVs reduced the number of TUNEL+ cells even at lower doses after only 8 h of treatment. In a final experiment, the authors found that EVs from NPCs were able to improve movement in a rodent model of cerebral ischemia to a similar level as treatment with the MSC-derived EVs. Impressively, this improvement persisted for 84 days post-stroke with an increase in neuronal density and proliferating cells. This approach further confirms the therapeutic utility of EVs and opens up new potential sources for producing functional neurons *in vivo*. Treatments could even be considered that induce endogenous NPCs to release EVs without the need for generating cells and EVs in the lab.

### Brain Cancer

The cancer stem cell hypothesis predates human pluripotent stem cell technology though still remains controversial (see Gimple et al., 2019 for a recent review). Methods for isolation and culture of CNS solid tumor stem cells are established and can be used for examining the first steps of cancer formation (see Goranci-Buzhala et al., 2021 for a recent example). Human stem cells (endogenous and PSC-derived) make excellent complimentary models for studying the cell of origin, cellular heterogeneity, and mechanisms of progression of multiple types of brain cancers, particularly since dedifferentiation and re-activation of an embryonic stem-like state is a hallmark of many cancers. This has been particularly true in efforts to model the highly lethal brain cancer glioblastoma multiforme (Gimple et al., 2019) but has also been used for non CNS cancers like medulloblastoma (Xue et al., 2021) and neuroblastoma (Cohen et al., 2020). Researchers have found that 3D models are particularly successful in modeling the lineage progression, cellular heterogeneity and invasive potential of these devastating cancers (see Luo and Li, 2021 for a recent review). 3D modeling approaches for glioblastoma are ever evolving and have included organoids generated from primary patient samples or hPSC derivatives, and more recently bioprinted constructs that aim to more faithfully recapitulate multiple elements of the tumor tissue microenvironment (Gimple et al., 2019; Zhang et al., 2020; Stanković et al., 2021). There are two main approaches to 3D cancer modeling: creating a “tumorsphere” consisting of cancer cells to model growth, signaling and drug response (Lenin et al., 2021; Pinto et al., 2021) or co-culture of cancer cells with “normal” organoids to study invasion and tumor formation (Choe et al., 2020; Azzarelli et al., 2021). Some groups have even reproduced tumor initiating events by introducing known tumor causing mutations into wild type organoids (Kim H. M. et al., 2021). In one compelling example, a streamlined strategy was recently developed to establish organoids from resected patient tumor tissues that closely recapitulate the cell type heterogeneity, histological patterns, mutation spectrum, and gene expression patterns of the parental tumors (Jacob et al., 2020). The authors created a biobanking strategy for these patient-derived models, and eloquently demonstrated their utility in modeling tumor invasiveness following mouse brain xenografts and in high throughput drug testing, including demonstrated sensitivity to CAR-T cell immunotherapy. The next phase of 3D cancer modeling will involve increasing the complexity of organoids to capture the tumor microenvironment (Ruiz-Garcia et al., 2020) by including key aspects such as vascularization and immune components (reviewed in Majc et al., 2021).

Medulloblastoma is the most common malignant brain tumor. Patients with Gorlin Syndrome, caused by a mutation in *PTCH1*, develop pediatric medulloblastomas at a rate of 5%. *PTCH1* mutations are also common in sporadic medulloblastoma cases, making Gorlin patients a useful model to study the mechanism of this gene in cancer initiation. Susanto et al. generated iPSC from a patient with a mutation leading to a truncation in PTCH1 protein and differentiated them, along with a control line, to neuroepithelial stem cells (NES cells) (Susanto et al., 2020). They found that patient NES cells showed a decrease in apoptosis compared to the unaffected control NES cells. When injected into the brains of mice only the patient, not control, NES cells grew into tumors with histology reflective of medulloblastoma. Secondary cells isolated from those tumors were able to form new tumors in a second injected mouse, confirming their transformation. These tumors showed expression of genes similar to a common SHH-dependent medulloblastoma type in humans and responded similarly to Vismodegib, a common treatment for SHH-subgroup medulloblastoma. Examining the transcriptional differences between the parent patient NES cells, and those from primary and secondary tumors revealed an increase in *LGALS1* expression, a gene which is novel in medulloblastoma but has been shown to promote invasiveness and immune evasion in neuroblastoma. Using stem cell derived neural cells to model cancer initiating events that reflect *in vivo* biology therefore has great utility for novel mechanistic and therapeutic discovery.

Stem cell derived models are also important tools to validate potential novel cancer drugs discovered in animal models. Atorvastatin is a potential cancer therapeutic that can cross the blood brain barrier; however the systemic dose needed for an effective dose in the brain is cytotoxic. Lübtow et al. (2020) created novel micellar atorvastatin formulations and tested their efficacy against established glioblastoma cell lines and cultured mouse tumor spheroids. The new formulations reduced the growth of both human and mouse cancer cells. To determine if the new formulation could cross the blood brain barrier, and at what concentration, the authors established an iPS cell-derived human blood brain barrier model in a transwell system. With this innovative approach they were able to administer high doses of the drug with no cytotoxic effects and found that the new formulations could cross their engineered blood brain barrier.

## The Next Steps in Human Cell Models

Cell replacement therapy is often discussed as one of the main applications of PSC-derived neural cells. Much work is being done to perfect differentiation protocols to achieve high yield, purity, functionality and regulatory compliance. Ganat et al. developed one of the first protocols for dopaminergic neuron differentiation to be used for cell replacement therapy for Parkinson's disease (Ganat et al., 2012) (see Parmar et al., 2019 for a recent review of Parkinson's Disease clinical trials). Autologous transplant, where a patient's tissue is reprogrammed and differentiated to the target cell type, is most certainly too costly and too slow for effective therapeutic intervention. Transdifferentiation (as mentioned above) has been a popular avenue of exploration in this regard. Several groups are pursuing transdifferentiation of astrocytes into neurons for cell replacement therapy (Brulet et al., 2017; Gao L. et al., 2017; Zhu et al., 2018; Zhao A. D. et al., 2020). Another approach is that of generating the “universal cell donor” where banks of genetically modified universal cell donor or homozygous HLA matched iPSCs are generated. This promising approach is still actively under development but offers substantial potential for long-term sustained impacts (Han et al., 2019).

Scale-up manufacture and purity of differentiated cultures are key considerations for cell replacement therapy, with labor and financial costs posing major hurdles to overcome (Kim and Kino-Oka, 2020). It will not be enough to be able to produce billions of cells simultaneously if no one can afford the cost to inject them into patients. One Phase I trial injected up to 20 million NPCs into stroke patients (Kalladka et al., 2016), while for other transplanted tissue types 1–10 × 10^9^ cells per patient are required (Kropp et al., 2017). While stem cells have a high proliferative potential, increased time in culture means more opportunity for genetic instability. Protocols have been developed for large scale iPSC generation and culture (Nathanson, 1989; Ismadi et al., 2014; Otsuji et al., 2014; Pagliuca et al., 2014). Bioreactor scale differentiation of PSCs into NPCs has also been developed (Miranda et al., 2015), providing a second intermediate proliferative cell type for expansion. Methods for cortical neurons generated in suspension culture (Parmet and Berg, 1989) and attachment-based scale up methods are also feasible in “cell-factory” style culture systems (Tirughana et al., 2018).

Toward disease modeling in a dish, brain organoids that mimic more diverse elements of tissue architecture are advancing rapidly. With the recent development of protocols to differentiate microglia, brain microvascular endothelial cells, pericytes, and choroid plexus and to then co-culture them with neural organoids, we are well on our way to building structures that truly mimic the *in vivo* cellular environment. Recent development of choroid plexus organoids allows for complementary blood-cerebrospinal fluid barrier modeling (Pellegrini et al., 2020). True vascularization of organoids is clearly a goal of the field, however, this has not yet been fully established in culture and has required transplant of engineered tissues into recipient animal models (Mansour et al., 2018). The most recent work in this field, however, has shown that co-culture of blood vessel organoids with neural organoids can create vascularization *in vitro* (Ahn et al., 2021). While an exciting advance, additional work will be needed to determine the functionality and long-term viability of these *in vitro* vessels.

Organ on a chip technology (Figure 2) allows for regulated flow through microfluidic channels and precise placement of cells in concert with them. This approach is especially promising for developing more complex brain neovascular unit models (Campisi et al., 2021). Blood brain barrier modeling is relevant to disease modeling (Pelkonen et al., 2020) and drug delivery studies (Wang X. et al., 2020). An added advantage over vascularized organoid models is the ability to control flow rate, pressure and compound delivery though the channels (see Lovett et al., 2020 for a review of organoids vs. bioengineered 3D models). Microfluidics has been used recently to examine barrier function upon SARS-CoV-2 infection (Buzhdygan et al., 2020) as well as to mimic signaling gradients to model neurodevelopment (Rifes et al., 2020). Outside of the brain, microfluidic models can be used to mimic flow through a variety of lumens and linking several tissue chips in succession is an intriguing way to model systemic interactions (see Raimondi et al., 2019 for a review on gut-brain-axis modeling).

The neural contribution of primarily non-CNS diseases can also now be examined given the breadth of stem cell-derived models that have recently been developed (Maiullari et al., 2018). Enteric neurons have been co-cultured with human intestinal organoids (Workman et al., 2017). Spinal motor neurons in co-culture with stem cell-derived muscle cells can form working neuromuscular junctions to permit analysis of processes underlying spinal muscular atrophy (Afshar Bakooshli et al., 2019). Sympathetic and parasympathetic neurons co-cultured with cardiac muscle can be used to test the effect of drugs on heart rate and heart health (Winbo et al., 2020). The next level of these complex co-cultures is 3D tissue printing (Figure 2). Using bio-mimic matrix type “inks” cells are layered into precise patterns in pre-set ratios and architectural patterns to accurately reproduce *in vivo* tissue structures. This technology has been used to create trachea (Gao M. et al., 2017), heart (Maiullari et al., 2018), and skin (Lee et al., 2014). Advances in 3D bioprinting of brain tissues are also on the rise with recent studies reported in modeling tumor, spinal cord, and developing cortex microenvironments (Walus et al., 2020). The advantage of bioprinted constructs is the level of control over spatial organization, potentially increasing the reproducibility over spontaneously formed organoids (see Li Y. C. et al., 2020 for an example). In a recent example, printed adipose-derived mesenchymal stem cells were differentiated into dopaminergic neurons displaying electrical response, a step toward functional patient-derived tissue for transplantation (Restan Perez et al., 2021). This technology is particularly suited to cancer modeling as normal brain tissue, cancer stem cells and associated immune cells can be positioned to mimic tumor growth, invasion, drug and immunological response (Tang et al., 2020). Incorporation of drug releasing microspheres into printed constructs can promote patterning during differentiation to generate tissue from multiple regions in the same construct (Sharma et al., 2020). Many of these tissue models now also incorporate microfluidic channels, providing advanced environmental control. With the ultimate goal of engineering functional transplantable tissues for therapeutics, this exciting technology paves the way for the production of rationally patterned heterogeneous full organs and tissue constructs entirely produced *in vitro* from human stem cells.

## Conclusion

The most robust neurological phenotypes and clinically impactful drug targets are those that are reproducible across multiple cell lines, biological tissue samples, animal models, and experimental techniques. The power of combining traditional cell culture and animal models with the emerging technologies described here, is the ability to deeply interrogate cellular mechanisms of development and disease and to then translate these findings in clinically relevant ways. The future of translational medicine research will increase the demand for validation in human-relevant models in addition to animal studies. Science done through collaboration between groups specializing in model organisms and those specializing in human stem cell culture will yield the most fruitful research to this end. A focus on the standardization of differentiation procedures and cost-efficient scale-up methods is necessary to drive these technologies closer to *in vivo* modeling and to unlock their full potential for cellular therapy and regenerative potential.

## Data Availability Statement

The original contributions presented in the study are included in the article/supplementary material, further inquiries can be directed to the corresponding author/s.

## Author Contributions

EK and LMJ contributed to the conceptualization, writing, and editing of this manuscript. All authors contributed to the article and approved the submitted version.

## Funding

LMJ is a Tier II Canada Research Chair and a Michael Smith Foundation for Health Research/Parkinson Society BC Scholar. She would also like to acknowledge funding support from: Simon Fraser University (start-up grants from the Faculty of Science and Department of Biological Sciences), the Cancer Research Society (JULIAN,L-CRS 25551), and the Natural Sciences and Engineering Research Council of Canada (JULIAN,L-RGPIN-03965).

## Conflict of Interest

EK is an employee of STEMCELL Technologies Canada Inc. The remaining author declares that the research was conducted in the absence of any commercial or financial relationships that could be construed as a potential conflict of interest.

## Publisher's Note

All claims expressed in this article are solely those of the authors and do not necessarily represent those of their affiliated organizations, or those of the publisher, the editors and the reviewers. Any product that may be evaluated in this article, or claim that may be made by its manufacturer, is not guaranteed or endorsed by the publisher.

## References

[B1] Abud E. M. Ramirez R. N. Martinez E. S. Healy L. M. Nguyen C. H. H. Newman S. A. . (2017). iPSC-derived human microglia-like cells to study neurological diseases. Neuron 94, 278–293.e279. 10.1016/j.neuron.2017.03.042 28426964 PMC5482419

[B2] Afshar Bakooshli M. Lippmann E. S. Mulcahy B. Iyer N. Nguyen C. T. Tung K. . (2019). A 3D culture model of innervated human skeletal muscle enables studies of the adult neuromuscular junction. Elife 8:e44530. 10.7554/eLife.44530.033 31084710 PMC6516829

[B3] Ahn Y. An J. H. Yang H. J. Lee D. G. Kim J. Koh H. . (2021). Human Blood Vessel Organoids Penetrate Human Cerebral Organoids And Form A Vessel-Like System. Cells 10:2036. 10.3390/cells10082036 34440805 PMC8393185

[B4] Anastasaki C. Wegscheid M. L. Hartigan K. Papke J. B. Kopp N. D. Chen J. . (2020). Human iPSC-derived neurons and cerebral organoids establish differential effects of germline NF1 gene mutations. Stem Cell Rep. 14, 541–550. 10.1016/j.stemcr.2020.03.007 32243842 PMC7160375

[B5] Andrews M. G. Subramanian L. Kriegstein A. R. (2020). mTOR signaling regulates the morphology and migration of outer radial glia in developing human cortex. Elife 9:e58737. 10.7554/eLife.58737.sa2 32876565 PMC7467727

[B6] Aravantinou-Fatorou K. Thomaidou D. (2020). *In vitro* direct reprogramming of mouse and human astrocytes to induced neurons. Methods Mol. Biol. 2155, 41–61. 10.1007/978-1-0716-0655-1_4 32474866

[B7] Arzua T. Yan Y. Jiang C. Logan S. Allison R. L. Wells C. . (2020). Modeling alcohol-induced neurotoxicity using human induced pluripotent stem cell-derived three-dimensional cerebral organoids. Transl. Psychiatry 10:347. 10.1038/s41398-020-01029-4 33051447 PMC7553959

[B8] Azevedo F. A. Carvalho L. R. Grinberg L. T. Farfel J. M. Ferretti R. E. Leite R. E. . (2009). Equal numbers of neuronal and nonneuronal cells make the human brain an isometrically scaled-up primate brain. J. Comp. Neurol. 513, 532–541. 10.1002/cne.21974 19226510

[B9] Azzarelli R. Ori M. Philpott A. Simons B. D. (2021). Three-dimensional model of glioblastoma by co-culturing tumor stem cells with human brain organoids. Biol. Open 10:bio056416. 10.1242/bio.056416 33619017 PMC7928227

[B10] Bagley J. A. Reumann D. Bian S. Lévi-Strauss J. Knoblich J. A. (2017). Fused cerebral organoids model interactions between brain regions. Nat. Methods 14, 743–751. 10.1038/nmeth.4304 28504681 PMC5540177

[B11] Baker D. E. Harrison N. J. Maltby E. Smith K. Moore H. D. Shaw P. J. . (2007). Adaptation to culture of human embryonic stem cells and oncogenesis *in vivo*. Nat. Biotechnol. 25, 207–215. 10.1038/nbt1285 17287758

[B12] Bakken T. E. Jorstad N. L. Hu Q. Lake B. B. Tian W. Kalmbach B. E. . (2021). Comparative cellular analysis of motor cortex in human, marmoset and mouse. Nature 598, 111–119. 10.1038/s41586-021-03465-8 34616062 PMC8494640

[B13] Balakrishnan A. Belfiore L. Chu T. H. Fleming T. Midha R. Biernaskie J. . (2020). Insights into the role and potential of schwann cells for peripheral nerve repair from studies of development and injury. Front. Mol. Neurosci. 13:608442. 10.3389/fnmol.2020.608442 33568974 PMC7868393

[B14] Bardy C. van den Hurk M. Eames T. Marchand C. Hernandez R. V. Kellogg M. . (2015). Neuronal medium that supports basic synaptic functions and activity of human neurons *in vitro*. Proc. Natl. Acad. Sci. U.S.A. 112, E2725–E2734. 10.1073/pnas.1504393112 25870293 PMC4443325

[B15] Barrell W. B. Griffin J. N. Harvey J. L. Danovi D. Beales P. Grigoriadis A. E. . (2019). Induction of neural crest stem cells from bardet-biedl syndrome patient derived hiPSCs. Front. Mol. Neurosci. 12:139. 10.3389/fnmol.2019.00139 31293383 PMC6598745

[B16] Ben-David U. Benvenisty N. (2011). The tumorigenicity of human embryonic and induced pluripotent stem cells. Nat. Rev. Cancer 11, 268–277. 10.1038/nrc3034 21390058

[B17] Benito-Kwiecinski S. Giandomenico S. L. Sutcliffe M. Riis E. S. Freire-Pritchett P. Kelava I. . (2021). An early cell shape transition drives evolutionary expansion of the human forebrain. Cell. 184, 2084–2102.e2019. 10.1016/j.cell.2021.02.050 33765444 PMC8054913

[B18] Berg J. Sorensen S. A. Ting J. T. Miller J. A. Chartrand T. Buchin A. . (2021). Human neocortical expansion involves glutamatergic neuron diversification. Nature 598, 151–158. 10.1038/s41586-021-03813-8 34616067 PMC8494638

[B19] Bershteyn M. Nowakowski T. J. Pollen A. A. Di Lullo E. Nene A. Wynshaw-Boris A. . (2017). Human iPSC-derived cerebral organoids model cellular features of lissencephaly and reveal prolonged mitosis of outer radial glia. Cell Stem Cell. 20, 435–449.e434. 10.1016/j.stem.2016.12.007 28111201 PMC5667944

[B20] Birey F. Andersen J. Makinson C. D. Islam S. Wei W. Huber N. . (2017). Assembly of functionally integrated human forebrain spheroids. Nature 545, 54–59. 10.1038/nature22330 28445465 PMC5805137

[B21] Blanchard J. W. Bula M. Davila-Velderrain J. Akay L. A. Zhu L. Frank A. . (2020). Reconstruction of the human blood-brain barrier *in vitro* reveals a pathogenic mechanism of APOE4 in pericytes. Nat. Med. 26, 952–963. 10.1038/s41591-020-0886-4 32514169 PMC7704032

[B22] Blum B. Benvenisty N. (2008). The tumorigenicity of human embryonic stem cells. Adv. Cancer Res. 100, 133–158. 10.1016/S0065-230X(08)00005-5 18620095

[B23] Brulet R. Matsuda T. Zhang L. Miranda C. Giacca M. Kaspar B. K. . (2017). NEUROD1 instructs neuronal conversion in non-reactive astrocytes. Stem Cell Rep. 8, 1506–1515. 10.1016/j.stemcr.2017.04.013 28506534 PMC5470076

[B24] Buzhdygan T. P. DeOre B. J. Baldwin-Leclair A. Bullock T. A. McGary H. M. Khan J. A. . (2020). The SARS-CoV-2 spike protein alters barrier function in 2D static and 3D microfluidic *in-vitro* models of the human blood-brain barrier. Neurobiol. Dis. 146:105131. 10.1016/j.nbd.2020.105131 33053430 PMC7547916

[B25] Campisi M. Lim S. H. Chiono V. Kamm R. D. (2021). 3D Self-organized human blood-brain barrier in a microfluidic chip. Methods Mol. Biol. 2258, 205–219. 10.1007/978-1-0716-1174-6_14 33340363

[B26] Cavalcante B. R. R. Aragão-França L. S. Sampaio G. L. A. Nonaka C. K. V. Oliveira M. S. Campos G. S. . (2020). Betulinic acid exerts cytoprotective activity on zika virus-infected neural progenitor cells. Front. Cell. Infect. Microbiol. 10:558324. 10.3389/fcimb.2020.558324 33251156 PMC7674920

[B27] Cederquist G. Y. Tchieu J. Callahan S. J. Ramnarine K. Ryan S. Zhang C. . (2020). A multiplex human pluripotent stem cell platform defines molecular and functional subclasses of autism-related genes. Cell Stem Cell 27, 35–49.e36. 10.1016/j.stem.2020.06.004 32619517 PMC7376579

[B28] Cenini G. Hebisch M. Iefremova V. Flitsch L. J. Breitkreuz Y. Tanzi R. E. . (2021). Dissecting Alzheimer's disease pathogenesis in human 2D and 3D models. Mol. Cell. Neurosci. 110:103568. 10.1016/j.mcn.2020.103568 33068718

[B29] Chambers S. M. Fasano C. A. Papapetrou E. P. Tomishima M. Sadelain M. Studer L. (2009). Highly efficient neural conversion of human ES and iPS cells by dual inhibition of SMAD signaling. Nat. Biotechnol. 27, 275–280. 10.1038/nbt.1529 19252484 PMC2756723

[B30] Chan W. K. Fetit R. Griffiths R. Marshall H. Mason J. O. Price D. J. (2021). Using organoids to study human brain development and evolution. Dev. Neurobiol. 81, 608–622. 10.1002/dneu.22819 33773072

[B31] Chen R. Wu X. Jiang L. Zhang Y. (2017). Single-Cell RNA-seq reveals hypothalamic cell diversity. Cell Rep. 18, 3227–3241. 10.1016/j.celrep.2017.03.004 28355573 PMC5782816

[B32] Choe M. S. Kim J. S. Yeo H. C. Bae C. M. Han H. J. Baek K. . (2020). A simple metastatic brain cancer model using human embryonic stem cell-derived cerebral organoids. FASEB J. 34, 16464–16475. 10.1096/fj.202000372R 33099835

[B33] Cohen M. A. Zhang S. Sengupta S. Ma H. Bell G. W. Horton B. . (2020). Formation of human neuroblastoma in mouse-human neural crest chimeras. Cell Stem Cell 26, 579–592.e576. 10.1016/j.stem.2020.02.001 32142683 PMC7663823

[B34] Commons K. G. Cholanians A. B. Babb J. A. Ehlinger D. G. (2017). The rodent forced swim test measures stress-coping strategy, not depression-like behavior. ACS Chem. Neurosci. 8, 955–960. 10.1021/acschemneuro.7b00042 28287253 PMC5518600

[B35] Corder E. H. Saunders A. M. Strittmatter W. J. Schmechel D. E. Gaskell P. C. Small G. W. . (1993). Gene dose of apolipoprotein E type 4 allele and the risk of Alzheimer's disease in late onset families. Science 261, 921–923. 10.1126/science.8346443 8346443

[B36] Cornacchia D. Studer L. (2017). Back and forth in time: directing age in iPSC-derived lineages. Brain Res. 1656, 14–26. 10.1016/j.brainres.2015.11.013 26592774 PMC4870156

[B37] Croft C. L. Futch H. S. Moore B. D. Golde T. E. (2019). Organotypic brain slice cultures to model neurodegenerative proteinopathies. Mol. Neurodegener. 14:45. 10.1186/s13024-019-0346-0 31791377 PMC6889333

[B38] Delaney S. P. Julian L. M. Pietrobon A. Yockell-Lelievre J. Dore C. Wang T. T. . (2020). Human pluripotent stem cell modeling of tuberous sclerosis complex reveals lineage-specific therapeutic vulnerabilities. bioRxiv. 1–83. 10.2139/ssrn.3554075

[B39] di Domenico A. Carola G. Calatayud C. Pons-Espinal M. Muñoz J. P. Richaud-Patin Y. . (2019). Patient-specific iPSC-derived astrocytes contribute to non-cell-autonomous neurodegeneration in Parkinson's disease. Stem Cell Rep. 12, 213–229. 10.1016/j.stemcr.2018.12.011 30639209 PMC6372974

[B40] Dos Reis R. S. Sant S. Keeney H. Wagner M. C. E. Ayyavoo V. (2020). Modeling HIV-1 neuropathogenesis using three-dimensional human brain organoids (hBORGs) with HIV-1 infected microglia. Sci. Rep. 10:15209. 10.1038/s41598-020-72214-0 32938988 PMC7494890

[B41] Douvaras P. Wang J. Zimmer M. Hanchuk S. O'Bara M. A. Sadiq S. . (2014). Efficient generation of myelinating oligodendrocytes from primary progressive multiple sclerosis patients by induced pluripotent stem cells. Stem Cell Rep. 3, 250–259. 10.1016/j.stemcr.2014.06.012 25254339 PMC4176529

[B42] D'Souza G. X. Rose S. E. Knupp A. Nicholson D. A. Keene C. D. Young J. E. (2021). The application of *in vitro*-derived human neurons in neurodegenerative disease modeling. J. Neurosci. Res. 99, 124–140. 10.1002/jnr.24615 32170790 PMC7487003

[B43] Duma C. Kopyov O. Kopyov A. Berman M. Lander E. Elam M. . (2019). Human intracerebroventricular (ICV) injection of autologous, non-engineered, adipose-derived stromal vascular fraction (ADSVF) for neurodegenerative disorders: results of a 3-year phase 1 study of 113 injections in 31 patients. Mol. Biol. Rep. 46, 5257–5272. 10.1007/s11033-019-04983-5 31327120

[B44] Eiraku M. Takata N. Ishibashi H. Kawada M. Sakakura E. Okuda S. . (2011). Self-organizing optic-cup morphogenesis in three-dimensional culture. Nature 472, 51–56. 10.1038/nature09941 21475194

[B45] El-Daher F. Becker C. G. (2020). Neural circuit reorganisation after spinal cord injury in zebrafish. Curr. Opin. Genet. Dev. 64, 44–51. 10.1016/j.gde.2020.05.017 32604009

[B46] Elfarrash S. Jensen N. M. Ferreira N. Betzer C. Thevathasan J. V. Diekmann R. . (2019). Organotypic slice culture model demonstrates inter-neuronal spreading of alpha-synuclein aggregates. Acta Neuropathol. Commun. 7:213. 10.1186/s40478-019-0865-5 31856920 PMC6924077

[B47] Enright H. A. Lam D. Sebastian A. Sales A. P. Cadena J. Hum N. R. . (2020). Functional and transcriptional characterization of complex neuronal co-cultures. Sci. Rep. 10:11007. 10.1038/s41598-020-67691-2 32620908 PMC7335084

[B48] Enver T. Soneji S. Joshi C. Brown J. Iborra F. Orntoft T. . (2005). Cellular differentiation hierarchies in normal and culture-adapted human embryonic stem cells. Hum. Mol. Genet. 14, 3129–3140. 10.1093/hmg/ddi345 16159889

[B49] Ernst C. (2020). A roadmap for neurodevelopmental disease modeling for non-stem cell biologists. Stem Cells Transl. Med. 9, 567–574. 10.1002/sctm.19-0344 32052596 PMC7180294

[B50] Esteban M. A. Wang T. Qin B. Yang J. Qin D. Cai J. . (2010). Vitamin C enhances the generation of mouse and human induced pluripotent stem cells. Cell Stem Cell 6, 71–79. 10.1016/j.stem.2009.12.001 20036631

[B51] Eura N. Matsui T. K. Luginbühl J. Matsubayashi M. Nanaura H. Shiota T. . (2020). Brainstem organoids from human pluripotent stem cells. Front. Neurosci. 14:538. 10.3389/fnins.2020.00538 32670003 PMC7332712

[B52] Eze U. C. Bhaduri A. Haeussler M. Nowakowski T. J. Kriegstein A. R. (2021). Single-cell atlas of early human brain development highlights heterogeneity of human neuroepithelial cells and early radial glia. Nat. Neurosci. 24, 584–594. 10.1038/s41593-020-00794-1 33723434 PMC8012207

[B53] Fair S. R. Julian D. Hartlaub A. M. Pusuluri S. T. Malik G. Summerfied T. L. . (2020). Electrophysiological maturation of cerebral organoids correlates with dynamic morphological and cellular development. Stem Cell Rep. 15, 855–868. 10.1016/j.stemcr.2020.08.017 32976764 PMC7562943

[B54] Falk A. Koch P. Kesavan J. Takashima Y. Ladewig J. Alexander M. . (2012). Capture of neuroepithelial-like stem cells from pluripotent stem cells provides a versatile system for *in vitro* production of human neurons. PLoS ONE 7:e29597. 10.1371/journal.pone.0029597 22272239 PMC3260177

[B55] Fang E. F. Hou Y. Palikaras K. Adriaanse B. A. Kerr J. S. Yang B. . (2019). Mitophagy inhibits amyloid-β and tau pathology and reverses cognitive deficits in models of Alzheimer's disease. Nat. Neurosci. 22, 401–412. 10.1038/s41593-018-0332-9 30742114 PMC6693625

[B56] Farzaneh M. Anbiyaiee A. Khoshnam S. E. (2020). Human pluripotent stem cells for spinal cord injury. Curr. Stem Cell Res. Ther. 15, 135–143. 10.2174/1574362414666191018121658 31656156

[B57] Fligor C. M. Langer K. B. Sridhar A. Ren Y. Shields P. K. Edler M. C. . (2018). Three-dimensional retinal organoids facilitate the investigation of retinal ganglion cell development, organization and neurite outgrowth from human pluripotent stem cells. Sci. Rep. 8:14520. 10.1038/s41598-018-32871-8 30266927 PMC6162218

[B58] Gabriel E. Ramani A. Karow U. Gottardo M. Natarajan K. Gooi L. M. . (2017). Recent Zika virus isolates induce premature differentiation of neural progenitors in human brain organoids. Cell Stem Cell 20, 397–406.e395. 10.1016/j.stem.2016.12.005 28132835

[B59] Ganat Y. M. Calder E. L. Kriks S. Nelander J. Tu E. Y. Jia F. . (2012). Identification of embryonic stem cell-derived midbrain dopaminergic neurons for engraftment. J. Clin. Invest. 122, 2928–2939. 10.1172/JCI58767 22751106 PMC3408729

[B60] Gao L. Guan W. Wang M. Wang H. Yu J. Liu Q. . (2017). Direct generation of human neuronal cells from adult astrocytes by small molecules. Stem Cell Rep. 8, 538–547. 10.1016/j.stemcr.2017.01.014 28216149 PMC5355633

[B61] Gao M. Zhang H. Dong W. Bai J. Gao B. Xia D. . (2017). Tissue-engineered trachea from a 3D-printed scaffold enhances whole-segment tracheal repair. Sci. Rep. 7:5246. 10.1038/s41598-017-05518-3 28701742 PMC5507982

[B62] García-León J. A. Kumar M. Boon R. Chau D. One J. Wolfs E. . (2018). SOX10 single transcription factor-based fast and efficient generation of oligodendrocytes from human pluripotent stem cells. Stem Cell Rep. 10, 655–672. 10.1016/j.stemcr.2017.12.014 29337119 PMC5830935

[B63] Ghanavatinejad F. Fard Tabrizi Z. P. Omidghaemi S. Sharifi E. Møller S. G. Jami M. S. (2019). Protein biomarkers of neural system. J. Otol. 14, 77–88. 10.1016/j.joto.2019.03.001 31467504 PMC6712353

[B64] Giandomenico S. L. Sutcliffe M. Lancaster M. A. (2021). Generation and long-term culture of advanced cerebral organoids for studying later stages of neural development. Nat. Protoc. 16, 579–602. 10.1038/s41596-020-00433-w 33328611 PMC7611064

[B65] Gimple R. C. Bhargava S. Dixit D. Rich J. N. (2019). Glioblastoma stem cells: lessons from the tumor hierarchy in a lethal cancer. Genes Dev. 33, 591–609. 10.1101/gad.324301.119 31160393 PMC6546059

[B66] Gomes A. R. Fernandes T. G. Vaz S. H. Silva T. P. Bekman E. P. Xapelli S. . (2020). Modeling Rett syndrome with human patient-specific forebrain organoids. Front. Cell Dev. Biol. 8:610427. 10.3389/fcell.2020.610427 33363173 PMC7758289

[B67] Gomez G. A. Prasad M. S. Sandhu N. Shelar P. B. Leung A. W. García-Castro M. I. (2019). Human neural crest induction by temporal modulation of WNT activation. Dev. Biol. 449, 99–106. 10.1016/j.ydbio.2019.02.015 30826399 PMC6685424

[B68] Goranci-Buzhala G. Mariappan A. Ricci-Vitiani L. Josipovic N. Pacioni S. Gottardo M. . (2021). Cilium induction triggers differentiation of glioma stem cells. Cell Rep. 36:109656. 10.1016/j.celrep.2021.109656 34496239

[B69] Gulinello M. Mitchell H. A. Chang Q. Timothy O'Brien W. Zhou Z. Abel T. . (2019). Rigor and reproducibility in rodent behavioral research. Neurobiol Learn Mem. 165:106780. 10.1016/j.nlm.2018.01.001 29307548 PMC6034984

[B70] Guo C. Pan Y. Gong Z. (2019). Recent advances in the genetic dissection of neural circuits in *Drosophila*. Neurosci. Bull. 35, 1058–1072. 10.1007/s12264-019-00390-9 31119647 PMC6864024

[B71] Hackland J. O. S. Frith T. J. R. Thompson O. Marin Navarro A. Garcia-Castro M. I. Unger C. . (2017). Top-down inhibition of BMP signaling enables robust induction of hPSCs into neural crest in fully defined, xeno-free conditions. Stem Cell Rep. 9, 1043–1052. 10.1016/j.stemcr.2017.08.008 28919261 PMC5639211

[B72] Halliwell J. Barbaric I. Andrews P. W. (2020). Acquired genetic changes in human pluripotent stem cells: origins and consequences. Nat. Rev. Mol. Cell Biol. 21, 715–728. 10.1038/s41580-020-00292-z 32968234

[B73] Han X. Wang M. Duan S. Franco P. J. Kenty J. H. Hedrick P. . (2019). Generation of hypoimmunogenic human pluripotent stem cells. Proc. Natl. Acad. Sci. U.S.A. 116, 10441–10446. 10.1073/pnas.1902566116 31040209 PMC6535035

[B74] Handel A. E. Chintawar S. Lalic T. Whiteley E. Vowles J. Giustacchini A. . (2016). Assessing similarity to primary tissue and cortical layer identity in induced pluripotent stem cell-derived cortical neurons through single-cell transcriptomics. Hum. Mol. Genet. 25, 989–1000. 10.1093/hmg/ddv637 26740550 PMC4754051

[B75] Henry M. P. Hawkins J. R. Boyle J. Bridger J. M. (2018). The genomic health of human pluripotent stem cells: genomic instability and the consequences on nuclear organization. Front. Genet. 9:623. 10.3389/fgene.2018.00623 30719030 PMC6348275

[B76] Hodge R. D. Bakken T. E. Miller J. A. Smith K. A. Barkan E. R. Graybuck L. T. . (2019). Conserved cell types with divergent features in human versus mouse cortex. Nature 573, 61–68. 10.1038/s41586-019-1506-7 31435019 PMC6919571

[B77] Höing S. Rudhard Y. Reinhardt P. Glatza M. Stehling M. Wu G. . (2012). Discovery of inhibitors of microglial neurotoxicity acting through multiple mechanisms using a stem-cell-based phenotypic assay. Cell Stem Cell 11, 620–632. 10.1016/j.stem.2012.07.005 23064101

[B78] Hor J. H. Ng S. Y. (2020). Generating ventral spinal organoids from human induced pluripotent stem cells. Methods Cell Biol. 159, 257–277. 10.1016/bs.mcb.2020.03.010 32586446

[B79] Hu B. Y. Zhang S. C. (2009). Differentiation of spinal motor neurons from pluripotent human stem cells. Nat. Protoc. 4, 1295–1304. 10.1038/nprot.2009.127 19696748 PMC2789120

[B80] Huangfu D. Osafune K. Maehr R. Guo W. Eijkelenboom A. Chen S. . (2008). Induction of pluripotent stem cells from primary human fibroblasts with only Oct4 and Sox2. Nat. Biotechnol. 26, 1269–1275. 10.1038/nbt.1502 18849973

[B81] Hubert C. G. Rivera M. Spangler L. C. Wu Q. Mack S. C. Prager B. C. . (2016). A three-dimensional organoid culture system derived from human glioblastomas recapitulates the hypoxic gradients and cancer stem cell heterogeneity of tumors found *in vivo*. Cancer Res. 76, 2465–2477. 10.1158/0008-5472.CAN-15-2402 26896279 PMC4873351

[B82] Huh C. J. Zhang B. Victor M. B. Dahiya S. Batista L. F. Horvath S. . (2016). Maintenance of age in human neurons generated by microRNA-based neuronal conversion of fibroblasts. Elife 5:e18648. 10.7554/eLife.18648.019 27644593 PMC5067114

[B83] Imaizumi K. Okano H. (2021). Modeling neurodevelopment in a dish with pluripotent stem cells. Dev. Growth Differ. 63, 18–25. 10.1111/dgd.12699 33141454 PMC7984205

[B84] Ismadi M. Z. Gupta P. Fouras A. Verma P. Jadhav S. Bellare J. . (2014). Flow characterization of a spinner flask for induced pluripotent stem cell culture application. PLoS ONE 9:e106493. 10.1371/journal.pone.0106493 25279733 PMC4184809

[B85] Jacob F. Salinas R. D. Zhang D. Y. Nguyen P. T. T. Schnoll J. G. Wong S. Z. H. . (2020). A patient-derived glioblastoma organoid model and biobank recapitulates inter- and intra-tumoral heterogeneity. Cell 180, 188–204.e122. 10.1016/j.cell.2019.11.036 31883794 PMC7556703

[B86] Jo J. Xiao Y. Sun A. X. Cukuroglu E. Tran H. D. Göke J. . (2016). Midbrain-like organoids from human pluripotent stem cells contain functional dopaminergic and neuromelanin-producing neurons. Cell Stem Cell 19, 248–257. 10.1016/j.stem.2016.07.005 27476966 PMC5510242

[B87] Kalladka D. Sinden J. Pollock K. Haig C. McLean J. Smith W. . (2016). Human neural stem cells in patients with chronic ischaemic stroke (PISCES): a phase 1, first-in-man study. Lancet 388, 787–796. 10.1016/S0140-6736(16)30513-X 27497862

[B88] Kamata Y. Isoda M. Sanosaka T. Shibata R. Ito S. Okubo T. . (2021). A robust culture system to generate neural progenitors with gliogenic competence from clinically relevant induced pluripotent stem cells for treatment of spinal cord injury. Stem Cells Transl. Med. 10, 398–413. 10.1002/sctm.20-0269 33226180 PMC7900588

[B89] Kanton S. Boyle M. J. He Z. Santel M. Weigert A. Sanchís-Calleja F. . (2019). Organoid single-cell genomic atlas uncovers human-specific features of brain development. Nature 574, 418–422. 10.1038/s41586-019-1654-9 31619793

[B90] Karzbrun E. Kshirsagar A. Cohen S. R. Hanna J. H. Reiner O. (2018). Human brain organoids on a chip reveal the physics of folding. Nat. Phys. 14, 515–522. 10.1038/s41567-018-0046-7 29760764 PMC5947782

[B91] Keynes R. Cook G. (2018). Segmentation of the chick central and peripheral nervous systems. Int. J. Dev. Biol. 62, 177–182. 10.1387/ijdb.170297rk 29616726

[B92] Kim H. M. Lee S. H. Lim J. Yoo J. Hwang D. Y. (2021). The epidermal growth factor receptor variant type III mutation frequently found in gliomas induces astrogenesis in human cerebral organoids. Cell Prolif. 54:e12965. 10.1111/cpr.12965 33283409 PMC7848959

[B93] Kim M. H. Kino-Oka M. (2020). Bioengineering considerations for a nurturing way to enhance scalable expansion of human pluripotent stem cells. Biotechnol. J. 15:e1900314. 10.1002/biot.201900314 31904180

[B94] Kim M. S. Kim D. H. Kang H. K. Kook M. G. Choi S. W. Kang K. S. (2021). Modeling of hypoxic brain injury through 3D human neural organoids. Cells 10:234. 10.3390/cells10020234 33504071 PMC7911731

[B95] Kirkeby A. Grealish S. Wolf D. A. Nelander J. Wood J. Lundblad M. . (2012). Generation of regionally specified neural progenitors and functional neurons from human embryonic stem cells under defined conditions. Cell Rep. 1, 703–714. 10.1016/j.celrep.2012.04.009 22813745

[B96] Krenn V. Bosone C. Burkard T. R. Spanier J. Kalinke U. Calistri A. . (2021). Organoid modeling of Zika and herpes simplex virus 1 infections reveals virus-specific responses leading to microcephaly. Cell Stem Cell. 28, 1362–1379.e1367. 10.1016/j.stem.2021.03.004 33838105 PMC7611471

[B97] Kropp C. Massai D. Zwiegerdt R. (2017). Progress and challenges in large-scale expansion of human pluripotent stem cells. Proc. Biochem. 59, 244–254. 10.1016/j.procbio.2016.09.032

[B98] Kuijlaars J. Oyelami T. Diels A. Rohrbacher J. Versweyveld S. Meneghello G. . (2016). Sustained synchronized neuronal network activity in a human astrocyte co-culture system. Sci. Rep. 6:36529. 10.1038/srep36529 27819315 PMC5098163

[B99] Lacalle-Aurioles M. Cassel de Camps C. Zorca C. E. Beitel L. K. Durcan T. M. (2020). Applying hiPSCs and biomaterials towards an understanding and treatment of traumatic brain injury. Front. Cell. Neurosci. 14:594304. 10.3389/fncel.2020.594304 33281561 PMC7689345

[B100] Lancaster M. A. Renner M. Martin C. A. Wenzel D. Bicknell L. S. Hurles M. E. . (2013). Cerebral organoids model human brain development and microcephaly. Nature 501, 373–379. 10.1038/nature12517 23995685 PMC3817409

[B101] Langston J. W. Ballard P. Tetrud J. W. Irwin I. (1983). Chronic Parkinsonism in humans due to a product of meperidine-analog synthesis. Science 219, 979–980. 10.1126/science.6823561 6823561

[B102] Laperle A. H. Sances S. Yucer N. Dardov V. J. Garcia V. J. Ho R. . (2020). iPSC modeling of young-onset Parkinson's disease reveals a molecular signature of disease and novel therapeutic candidates. Nat. Med. 26, 289–299. 10.1038/s41591-019-0739-1 31988461

[B103] Lee V. Singh G. Trasatti J. P. Bjornsson C. Xu X. Tran T. N. . (2014). Design and fabrication of human skin by three-dimensional bioprinting. Tissue Eng. Part C Methods 20, 473–484. 10.1089/ten.tec.2013.0335 24188635 PMC4024844

[B104] Lenin S. Ponthier E. Scheer K. G. Yeo E. C. F. Tea M. N. Ebert L. M. . (2021). A drug screening pipeline using 2D and 3D patient-derived *in vitro* models for pre-clinical analysis of therapy response in glioblastoma. Int. J. Mol. Sci. 22:4322. 10.3390/ijms22094322 33919246 PMC8122466

[B105] Leung A. W. Murdoch B. Salem A. F. Prasad M. S. Gomez G. A. García-Castro M. I. (2016). WNT/β-catenin signaling mediates human neural crest induction via a pre-neural border intermediate. Development 143, 398–410. 10.1242/dev.130849 26839343 PMC4760313

[B106] Leventoux N. Morimoto S. Imaizumi K. Sato Y. Takahashi S. Mashima K. . (2020). Human astrocytes model derived from induced pluripotent stem cells. Cells 9:2680. 10.3390/cells9122680 33322219 PMC7763297

[B107] Li J. Narayanan C. Bian J. Sambo D. Brickler T. Zhang W. . (2018). A transient DMSO treatment increases the differentiation potential of human pluripotent stem cells through the Rb family. PLoS ONE 13:e0208110. 10.1371/journal.pone.0208110 30540809 PMC6291069

[B108] Li X. Tao Y. Bradley R. Du Z. Kong L. Dong Y. . (2018). Fast generation of functional subtype astrocytes from human pluripotent stem cells. Stem Cell Rep. 11, 998–1008. 10.1016/j.stemcr.2018.08.019 30269954 PMC6178885

[B109] Li X. Xu R. Tu X. Janairo R. R. R. Kwong G. Wang D. . (2020). Differentiation of neural crest stem cells in response to matrix stiffness and TGF-β1 in vascular regeneration. Stem Cells Dev. 29, 249–256. 10.1089/scd.2019.0161 31701817

[B110] Li Y. Muffat J. Omer A. Bosch I. Lancaster M. A. Sur M. . (2017). Induction of expansion and folding in human cerebral organoids. Cell Stem Cell 20, 385–396.e383. 10.1016/j.stem.2016.11.017 28041895 PMC6461394

[B111] Li Y. C. Jodat Y. A. Samanipour R. Zorzi G. Zhu K. Hirano M. . (2020). Toward a neurospheroid Niche model: optimizing embedded 3D bioprinting for fabrication of neurospheroid brain-like co-culture constructs. Biofabrication. 20:385–386. 10.1088/1758-5090/abc1be 33059333 PMC8387028

[B112] Libby A. R. G. Joy D. A. Elder N. H. Bulger E. A. Krakora M. Z. Gaylord E. A. . (2021). Axial elongation of caudalized human organoids mimics aspects of neural tube development. Development 148:dev.198275. 10.1242/dev.198275 34142711 PMC8254868

[B113] Lin B. McLelland B. T. Aramant R. B. Thomas B. B. Nistor G. Keirstead H. S. . (2020). Retina organoid transplants develop photoreceptors and improve visual function in RCS rats with RPE dysfunction. Invest. Ophthalmol. Vis. Sci. 61:34. 10.1167/iovs.61.11.34 32945842 PMC7509771

[B114] Liu T. Zhu B. Liu Y. Zhang X. Yin J. Li X. . (2020). Multi-omic comparison of Alzheimer's variants in human ESC-derived microglia reveals convergence at APOE. J. Exp. Med. 217:e20200474. 10.1084/jem.20200474 32941599 PMC7953740

[B115] Liu Y. Miao Q. Yuan J. Han S. Zhang P. Li S. . (2015). Ascl1 converts dorsal midbrain astrocytes into functional neurons *in vivo*. J. Neurosci. 35, 9336–9355. 10.1523/JNEUROSCI.3975-14.2015 26109658 PMC6605193

[B116] Livesey M. R. Magnani D. Hardingham G. E. Chandran S. Wyllie D. J. (2016). Functional properties of *in vitro* excitatory cortical neurons derived from human pluripotent stem cells. J. Physiol. 594, 6573–6582. 10.1113/JP270660 26608229 PMC5108911

[B117] Logan S. Arzua T. Yan Y. Jiang C. Liu X. Yu L. K. . (2020). Dynamic characterization of structural, molecular, and electrophysiological phenotypes of human-induced pluripotent stem cell-derived cerebral organoids, and comparison with fetal and adult gene profiles. Cells 9:1301. 10.3390/cells9051301 32456176 PMC7291286

[B118] Lovett M. L. Nieland T. J. F. Dingle Y. L. Kaplan D. L. (2020). Innovations in 3-dimensional tissue models of human brain physiology and diseases. Adv. Funct. Mater. 30:1909146. 10.1002/adfm.201909146 34211358 PMC8240470

[B119] Lu J. Zhong X. Liu H. Hao L. Huang C. T. Sherafat M. A. . (2016). Generation of serotonin neurons from human pluripotent stem cells. Nat. Biotechnol. 34, 89–94. 10.1038/nbt.3435 26655496 PMC4711820

[B120] Lübtow M. M. Oerter S. Quader S. Jeanclos E. Cubukova A. Krafft M. . (2020). Blood-brain barrier permeability and cytotoxicity of an atorvastatin-loaded nanoformulation against glioblastoma in 2D and 3D models. Mol. Pharm. 17, 1835–1847. 10.1021/acs.molpharmaceut.9b01117 32315193

[B121] Luo J. Li P. (2021). Human pluripotent stem cell-derived brain organoids as *in vitro* models for studying neural disorders and cancer. Cell Biosci. 11:99. 10.1186/s13578-021-00617-1 34049587 PMC8161602

[B122] Lutz B. Schmid W. Niehrs C. Schütz G. (1999). Essential role of CREB family proteins during Xenopus embryogenesis. Mech. Dev. 88, 55–66. 10.1016/S0925-4773(99)00170-7 10525188

[B123] Ma Y. Xie H. Du X. Wang L. Jin X. Zhang Q. . (2021). *In vivo* chemical reprogramming of astrocytes into neurons. Cell Discov. 7:12. 10.1038/s41421-021-00243-8 33649311 PMC7921425

[B124] Maiullari F. Costantini M. Milan M. Pace V. Chiriv,ì M. Maiullari S. . (2018). A multi-cellular 3D bioprinting approach for vascularized heart tissue engineering based on HUVECs and iPSC-derived cardiomyocytes. Sci. Rep. 8:13532. 10.1038/s41598-018-31848-x 30201959 PMC6131510

[B125] Majc B. Novak M. Kopitar-Jerala N. Jewett A. Breznik B. (2021). Immunotherapy of glioblastoma: current strategies and challenges in tumor model development. Cells 10:265. 10.3390/cells10020265 33572835 PMC7912469

[B126] Malankhanova T. Suldina L. Grigor'eva E. Medvedev S. Minina J. Morozova K. . (2020). A human induced pluripotent stem cell-derived isogenic model of huntington's disease based on neuronal cells has several relevant phenotypic abnormalities. J. Pers. Med. 10:215. 10.3390/jpm10040215 33182269 PMC7712151

[B127] Mansour A. A. Gonçalves J. T. Bloyd C. W. Li H. Fernandes S. Quang D. . (2018). An *in vivo* model of functional and vascularized human brain organoids. Nat. Biotechnol. 36, 432–441. 10.1038/nbt.4127 29658944 PMC6331203

[B128] Marcatili M. Sala C. Dakanalis A. Colmegna F. D'Agostino A. Gambini O. . (2020). Human induced pluripotent stem cells technology in treatment resistant depression: novel strategies and opportunities to unravel ketamine's fast-acting antidepressant mechanisms. Ther. Adv. Psychopharmacol. 10:2045125320968331. 10.1177/2045125320968331 33224469 PMC7649879

[B129] Marton R. M. Miura Y. Sloan S. A. Li Q. Revah O. Levy R. J. . (2019). Differentiation and maturation of oligodendrocytes in human three-dimensional neural cultures. Nat. Neurosci. 22, 484–491. 10.1038/s41593-018-0316-9 30692691 PMC6788758

[B130] Marton R. M. Paşca S. P. (2020). Organoid and assembloid technologies for investigating cellular crosstalk in human brain development and disease. Trends Cell Biol. 30, 133–143. 10.1016/j.tcb.2019.11.004 31879153

[B131] Matsui T. K. Tsuru Y. Kuwako K. I. (2020). Challenges in modeling human neural circuit formation via brain organoid technology. Front. Cell. Neurosci. 14:607399. 10.3389/fncel.2020.607399 33362473 PMC7756199

[B132] McQuade A. Coburn M. Tu C. H. Hasselmann J. Davtyan H. Blurton-Jones M. (2018). Development and validation of a simplified method to generate human microglia from pluripotent stem cells. Mol. Neurodegener. 13:67. 10.1186/s13024-018-0297-x 30577865 PMC6303871

[B133] McQuade A. Kang Y. J. Hasselmann J. Jairaman A. Sotelo A. Coburn M. . (2020). Gene expression and functional deficits underlie TREM2-knockout microglia responses in human models of Alzheimer's disease. Nat. Commun. 11:5370. 10.1038/s41467-020-19227-5 33097708 PMC7584603

[B134] Mertens J. Herdy J. R. Traxler L. Schafer S. T. Schlachetzki J. C. M. Böhnke L. . (2021). Age-dependent instability of mature neuronal fate in induced neurons from Alzheimer's patients. Cell Stem Cell. 28, 1533–1548.e1536. 10.1016/j.stem.2021.04.004 33910058 PMC8423435

[B135] Mertens J. Reid D. Lau S. Kim Y. Gage F. H. (2018). Aging in a dish: iPSC-derived and directly induced neurons for studying brain aging and age-related neurodegenerative diseases. Annu. Rev. Genet. 52, 271–293. 10.1146/annurev-genet-120417-031534 30208291 PMC6415910

[B136] Miller J. D. Ganat Y. M. Kishinevsky S. Bowman R. L. Liu B. Tu E. Y. . (2013). Human iPSC-based modeling of late-onset disease via progerin-induced aging. Cell Stem Cell. 13, 691–705. 10.1016/j.stem.2013.11.006 24315443 PMC4153390

[B137] Miranda C. C. Fernandes T. G. Pascoal J. F. Haupt S. Brüstle O. Cabral J. M. . (2015). Spatial and temporal control of cell aggregation efficiently directs human pluripotent stem cells towards neural commitment. Biotechnol. J. 10, 1612–1624. 10.1002/biot.201400846 25866360

[B138] Mitchell T. Archer D. B. Chu W. T. Coombes S. A. Lai S. Wilkes B. J. . (2019). Neurite orientation dispersion and density imaging (NODDI) and free-water imaging in Parkinsonism. Hum. Brain Mapp. 40, 5094–5107. 10.1002/hbm.24760 31403737 PMC6865390

[B139] Muguruma K. Nishiyama A. Kawakami H. Hashimoto K. Sasai Y. (2015). Self-organization of polarized cerebellar tissue in 3D culture of human pluripotent stem cells. Cell Rep. 10, 537–550. 10.1016/j.celrep.2014.12.051 25640179

[B140] Nathanson B. (1989). Operation rescue: domestic terrorism or legitimate civil rights protest? Hastings Cent. Rep. 19, 28–32. 10.2307/3561985 2606657

[B141] Naujock M. Speidel A. Fischer S. Kizner V. Dorner-Ciossek C. Gillardon F. (2020). Neuronal differentiation of induced pluripotent stem cells from schizophrenia patients in two-dimensional and in three-dimensional cultures reveals increased expression of the Kv4.2 subunit DPP6 that contributes to decreased neuronal activity. Stem Cells Dev. 29, 1577–1587. 10.1089/scd.2020.0082 33143549

[B142] Nehme R. Zuccaro E. Ghosh S. D. Li C. Sherwood J. L. Pietilainen O. . (2018). Combining NGN2 programming with developmental patterning generates human excitatory neurons with NMDAR-mediated synaptic transmission. Cell Rep. 23, 2509–2523. 10.1016/j.celrep.2018.04.066 29791859 PMC6003669

[B143] Nieves M. D. Furmanski O. Doughty M. L. (2020). Host sex and transplanted human induced pluripotent stem cell phenotype interact to influence sensorimotor recovery in a mouse model of cortical contusion injury. Brain Res. 1748:147120. 10.1016/j.brainres.2020.147120 32926852

[B144] Nolbrant S. Heuer A. Parmar M. Kirkeby A. (2017). Generation of high-purity human ventral midbrain dopaminergic progenitors for *in vitro* maturation and intracerebral transplantation. Nat. Protoc. 12, 1962–1979. 10.1038/nprot.2017.078 28858290

[B145] Odawara A. Saitoh Y. Alhebshi A. H. Gotoh M. Suzuki I. (2014). Long-term electrophysiological activity and pharmacological response of a human induced pluripotent stem cell-derived neuron and astrocyte co-culture. Biochem. Biophys. Res. Commun. 443, 1176–1181. 10.1016/j.bbrc.2013.12.142 24406164

[B146] Okita K. Yamakawa T. Matsumura Y. Sato Y. Amano N. Watanabe A. . (2013). An efficient nonviral method to generate integration-free human-induced pluripotent stem cells from cord blood and peripheral blood cells. Stem Cells 31, 458–466. 10.1002/stem.1293 23193063

[B147] Ortmann D. Vallier L. (2017). Variability of human pluripotent stem cell lines. Curr. Opin. Genet. Dev. 46, 179–185. 10.1016/j.gde.2017.07.004 28843810

[B148] Otsuji T. G. Bin J. Yoshimura A. Tomura M. Tateyama D. Minami I. . (2014). A 3D sphere culture system containing functional polymers for large-scale human pluripotent stem cell production. Stem Cell Rep. 2, 734–745. 10.1016/j.stemcr.2014.03.012 24936458 PMC4050473

[B149] Pagliuca F. W. Millman J. R. Gürtler M. Segel M. Van Dervort A. Ryu J. H. . (2014). Generation of functional human pancreatic β cells *in vitro*. Cell. 159, 428–439. 10.1016/j.cell.2014.09.040 25303535 PMC4617632

[B150] Park T. S. Zimmerlin L. Evans-Moses R. Zambidis E. T. (2018). Chemical reversion of conventional human pluripotent stem cells to a naïve-like state with improved multilineage differentiation potency. J. Vis. Exp. e57921:1–12. 10.3791/57921 29939183 PMC6101693

[B151] Parmar M. Torper O. Drouin-Ouellet J. (2019). Cell-based therapy for Parkinson's disease: a journey through decades toward the light side of the Force. Eur. J. Neurosci. 49, 463–471. 10.1111/ejn.14109 30099795 PMC6519227

[B152] Parmet A. J. Berg J. L. (1989). Cases from the aerospace medicine residents' teaching file. Case #31. A special operations person with methemoglobinemia is discussed, with attention to operation of nuclear weapons. Aviat. Space Environ. Med. 60, 465–466. 2730492

[B153] Pelkonen A. Mzezewa R. Sukki L. Ryynänen T. Kreutzer J. Hyvärinen T. . (2020). A modular brain-on-a-chip for modelling epileptic seizures with functionally connected human neuronal networks. Biosens. Bioelectron. 168:112553. 10.1016/j.bios.2020.112553 32877779

[B154] Pellegrini L. Bonfio C. Chadwick J. Begum F. Skehel M. Lancaster M. A. (2020). Human CNS barrier-forming organoids with cerebrospinal fluid production. Science 369:eaaz5626. 10.1126/science.aaz5626 32527923 PMC7116154

[B155] Pettke A. Tampere M. Pronk R. Wallner O. Falk A. Warpman Berglund U. . (2020). Broadly active antiviral compounds disturb zika virus progeny release rescuing virus-induced toxicity in brain organoids. Viruses 13:37. 10.3390/v13010037 33383826 PMC7823652

[B156] Piao J. Zabierowski S. Dubose B. N. Hill E. J. Navare M. Claros N. . (2021). Preclinical efficacy and safety of a human embryonic stem cell-derived midbrain dopamine progenitor product, MSK-DA01. Cell Stem Cell 28, 217–229.e217. 10.1016/j.stem.2021.01.004 33545080 PMC7903922

[B157] Pinto G. Saenz-de-Santa-Maria I. Chastagner P. Perthame E. Delmas C. Toulas C. . (2021). Patient-derived glioblastoma stem cells transfer mitochondria through tunneling nanotubes in tumor organoids. Biochem J. 478, 21–39. 10.1042/BCJ20200710 33245115 PMC7800365

[B158] Polepalli J. S. Gooch H. Sah P. (2020). Diversity of interneurons in the lateral and basal amygdala. NPJ Sci. Learn. 5:10. 10.1038/s41539-020-0071-z 32802405 PMC7400739

[B159] Pollen A. A. Nowakowski T. J. Chen J. Retallack H. Sandoval-Espinosa C. Nicholas C. R. . (2015). Molecular identity of human outer radial glia during cortical development. Cell 163, 55–67. 10.1016/j.cell.2015.09.004 26406371 PMC4583716

[B160] Prince L. M. Aschner M. Bowman A. B. (2019). Human-induced pluripotent stems cells as a model to dissect the selective neurotoxicity of methylmercury. Biochim. Biophys. Acta Gen. Subj. 1863:129300. 10.1016/j.bbagen.2019.02.002 30742955 PMC6689259

[B161] Prince M. Szer J. van der Weyden M. B. Pedersen J. S. Holdsworth R. F. Whyte G. (1991). Transfusion associated graft-versus-host disease after cardiac surgery: response to antithymocyte-globulin and corticosteroid therapy. Aust. N. Z. J. Med. 21, 43–46. 10.1111/j.1445-5994.1991.tb03000.x 2036076

[B162] Prytkova I. Brennand K. J. (2017). Prospects for modeling abnormal neuronal function in schizophrenia using human induced pluripotent stem cells. Front. Cell. Neurosci. 11:360. 10.3389/fncel.2017.00360 29217999 PMC5703699

[B163] Qian C. Dong B. Wang X. Y. Zhou F. Q. (2021). *In vivo* glial trans-differentiation for neuronal replacement and functional recovery in central nervous system. FEBS J. 288, 4773–4785. 10.1111/febs.15681 33351267 PMC8217397

[B164] Qian T. Maguire S. E. Canfield S. G. Bao X. Olson W. R. Shusta E. V. . (2017). Directed differentiation of human pluripotent stem cells to blood-brain barrier endothelial cells. Sci. Adv. 3:e1701679. 10.1126/sciadv.1701679 29134197 PMC5677350

[B165] Raimondi I. Izzo L. Tunesi M. Comar M. Albani D. Giordano C. (2019). Organ-On-A-Chip. Front Bioeng Biotechnol. 7:435. 10.3389/fbioe.2019.00435 31998702 PMC6965718

[B166] Ransohoff R. M. (2018). All (animal) models (of neurodegeneration) are wrong. Are they also useful? J. Exp. Med. 215, 2955–2958. 10.1084/jem.20182042 30459159 PMC6279414

[B167] Real R. Peter M. Trabalza A. Khan S. Smith M. A. Dopp J. . (2018). *In vivo* modeling of human neuron dynamics and Down syndrome. Science 362:eaau1810. 10.1126/science.aau1810 30309905 PMC6570619

[B168] Rehbach K. Fernando M. B. Brennand K. J. (2020). Integrating CRISPR engineering and hiPSC-derived 2D disease modeling systems. J. Neurosci. 40, 1176–1185. 10.1523/JNEUROSCI.0518-19.2019 32024766 PMC7002154

[B169] Restan Perez M. Sharma R. Masri N. Z. Willerth S. M. (2021). 3D Bioprinting mesenchymal stem cell-derived neural tissues using a fibrin-based bioink. Biomolecules 11:1250. 10.3390/biom11081250 34439916 PMC8394541

[B170] Riemens R. J. M. Kenis G. van den Beucken T. (2020). Human-induced pluripotent stem cells as a model for studying sporadic Alzheimer's disease. Neurobiol. Learn. Mem. 175:107318. 10.1016/j.nlm.2020.107318 32977028

[B171] Rifes P. Isaksson M. Rathore G. S. Aldrin-Kirk P. Møller O. K. Barzaghi G. . (2020). Modeling neural tube development by differentiation of human embryonic stem cells in a microfluidic WNT gradient. Nat. Biotechnol. 38, 1265–1273. 10.1038/s41587-020-0525-0 32451506 PMC7616963

[B172] Ruiz-Garcia H. Alvarado-Estrada K. Schiapparelli P. Quinones-Hinojosa A. Trifiletti D. M. (2020). Engineering three-dimensional tumor models to study glioma cancer stem cells and tumor microenvironment. Front. Cell. Neurosci. 14:558381. 10.3389/fncel.2020.558381 33177991 PMC7596188

[B173] Sabitha K. R. Shetty A. K. Upadhya D. (2021). Patient-derived iPSC modeling of rare neurodevelopmental disorders: molecular pathophysiology and prospective therapies. Neurosci. Biobehav. Rev. 121, 201–219. 10.1016/j.neubiorev.2020.12.025 33370574 PMC7962756

[B174] Sarkar A. Mei A. Paquola A. C. M. Stern S. Bardy C. Klug J. R. . (2018). Efficient generation of CA3 neurons from human pluripotent stem cells enables modeling of hippocampal connectivity *in vitro*. Cell Stem Cell 22, 684-697.e689. 10.1016/j.stem.2018.04.009 29727680 PMC6345574

[B175] Sawada T. Chater T. E. Sasagawa Y. Yoshimura M. Fujimori-Tonou N. Tanaka K. . (2020). Developmental excitation-inhibition imbalance underlying psychoses revealed by single-cell analyses of discordant twins-derived cerebral organoids. Mol. Psychiatry 25, 2695–2711. 10.1038/s41380-020-0844-z 32764691 PMC7577852

[B176] Schuster J. Halvardson J. Pilar Lorenzo L. Ameur A. Sobol M. Raykova D. . (2015). Transcriptome profiling reveals degree of variability in induced pluripotent stem cell lines: impact for human disease modeling. Cell. Reprog. 17, 327–337. 10.1089/cell.2015.0009 26348590

[B177] Shao Z. Noh H. Bin Kim W. Ni P. Nguyen C. Cote S. E. . (2019). Dysregulated protocadherin-pathway activity as an intrinsic defect in induced pluripotent stem cell-derived cortical interneurons from subjects with schizophrenia. Nat. Neurosci. 22, 229–242. 10.1038/s41593-018-0313-z 30664768 PMC6373728

[B178] Sharma R. Smits I. P. M. De La Vega L. Lee C. Willerth S. M. (2020). 3D Bioprinting Pluripotent stem cell derived neural tissues using a novel fibrin bioink containing drug releasing microspheres. Front Bioeng Biotechnol. 8:57. 10.3389/fbioe.2020.00057 32117936 PMC7026266

[B179] Shen H. H. (2016). News feature: better models for brain disease. Proc. Natl. Acad. Sci. U.S.A. 113, 5461–5464. 10.1073/pnas.1605358113 27190074 PMC4878480

[B180] Shi W. Dong P. Kuss M. A. Gu L. Kievit F. Kim H. J. . (2021). Design and evaluation of an *in vitro* mild traumatic brain injury modeling system using 3D printed mini impact device on the 3D cultured human iPSC derived neural progenitor cells. Adv. Healthc. Mater. 10:e2100180. 10.1002/adhm.202100180 33890428 PMC8222191

[B181] Shiga Y. Shiga A. Mesci P. Kwon H. Brifault C. Kim J. H. . (2019). Tissue-type plasminogen activator-primed human iPSC-derived neural progenitor cells promote motor recovery after severe spinal cord injury. Sci. Rep. 9:19291. 10.1038/s41598-019-55132-8 31848365 PMC6917728

[B182] Singh J. Aballay A. (2020). Neural control of behavioral and molecular defenses in *C. elegans*. Curr. Opin. Neurobiol. 62, 34–40. 10.1016/j.conb.2019.10.012 31812835 PMC7272302

[B183] Sloan S. A. Andersen J. Paşca A. M. Birey F. Paşca S. P. (2018). Generation and assembly of human brain region-specific three-dimensional cultures. Nat. Protoc. 13, 2062–2085. 10.1038/s41596-018-0032-7 30202107 PMC6597009

[B184] Smith J. A. Nicaise A. M. Ionescu R. B. Hamel R. Peruzzotti-Jametti L. Pluchino S. (2021). Stem cell therapies for progressive multiple sclerosis. Front. Cell Dev. Biol. 9:696434. 10.3389/fcell.2021.696434 34307372 PMC8299560

[B185] Sridharan B. Hubbs C. Llamosas N. Kilinc M. Singhera F. U. Willems E. . (2019). A simple procedure for creating scalable phenotypic screening assays in human neurons. Sci. Rep. 9:9000. 10.1038/s41598-019-45265-1 31227747 PMC6588600

[B186] Stanković T. Randelović T. Dragoj M. Stojković Burić S. Fernández L. Ochoa I. . (2021). *In vitro* biomimetic models for glioblastoma-a promising tool for drug response studies. Drug Resist. Updat. 55:100753. 10.1016/j.drup.2021.100753 33667959

[B187] Stebbins M. J. Gastfriend B. D. Canfield S. G. Lee M. S. Richards D. Faubion M. G. . (2019). Human pluripotent stem cell-derived brain pericyte-like cells induce blood-brain barrier properties. Sci. Adv. 5:eaau7375. 10.1126/sciadv.aau7375 30891496 PMC6415958

[B188] Sullivan S. Stacey G. N. Akazawa C. Aoyama N. Baptista R. Bedford P. . (2018). Quality control guidelines for clinical-grade human induced pluripotent stem cell lines. Regen. Med. 13, 859–866. 10.2217/rme-2018-0095 30205750

[B189] Susanto E. Marin Navarro A. Zhou L. Sundström A. van Bree N. Stantic M. . (2020). Modeling SHH-driven medulloblastoma with patient iPS cell-derived neural stem cells. Proc. Natl. Acad. Sci. U.S.A. 117, 20127–20138. 10.1073/pnas.1920521117 32747535 PMC7443968

[B190] Swierkosz T. A. Jordan L. McBride M. McGough K. Devlin J. Botting R. M. (2002). Actions of paracetamol on cyclooxygenases in tissue and cell homogenates of mouse and rabbit. Med Sci Monit. 8:BR496-503. 12503027

[B191] Takahashi J. (2020). iPS cell-based therapy for Parkinson's disease: a Kyoto trial. Regen. Ther. 13, 18–22. 10.1016/j.reth.2020.06.002 33490319 PMC7794047

[B192] Tang M. Xie Q. Gimple R. C. Zhong Z. Tam T. Tian J. . (2020). Three-dimensional bioprinted glioblastoma microenvironments model cellular dependencies and immune interactions. Cell Res. 30, 833–853. 10.1038/s41422-020-0338-1 32499560 PMC7608409

[B193] Tayanloo-Beik A. Rabbani Z. Soveyzi F. Alavi-Moghadam S. Rezaei-Tavirani M. Goodarzi P. . (2021). Cellular therapy for treatment of spinal cord injury in Zebrafish model. Mol. Biol. Rep. 48, 1787–1800. 10.1007/s11033-020-06126-7 33459959

[B194] Tchieu J. Zimmer B. Fattahi F. Amin S. Zeltner N. Chen S. . (2017). A modular platform for differentiation of human PSCs into all major ectodermal lineages. Cell Stem Cell 21, 399–410.e397. 10.1016/j.stem.2017.08.015 28886367 PMC5737635

[B195] Tcw J. Wang M. Pimenova A. A. Bowles K. R. Hartley B. J. Lacin E. . (2017). An Efficient platform for astrocyte differentiation from human induced pluripotent stem cells. Stem Cell Rep. 9, 600–614. 10.1016/j.stemcr.2017.06.018 28757165 PMC5550034

[B196] Tian A. Muffat J. Li Y. (2020). Studying human neurodevelopment and diseases using 3D brain organoids. J Neurosci. 40, 1186–1193. 10.1523/JNEUROSCI.0519-19.2019 32024767 PMC7002141

[B197] Tirughana R. Metz M. Z. Li Z. Hall C. Hsu D. Beltzer J. . (2018). GMP production and scale-up of adherent neural stem cells with a quantum cell expansion system. Mol. Ther. Methods Clin. Dev. 10, 48–56. 10.1016/j.omtm.2018.05.006 29992178 PMC6037686

[B198] Trujillo C. A. Gao R. Negraes P. D. Gu J. Buchanan J. Preissl S. . (2019). Complex oscillatory waves emerging from cortical organoids model early human brain network development. Cell Stem Cell 25, 558–569.e557. 10.1016/j.stem.2019.08.002 31474560 PMC6778040

[B199] Valadez-Barba V. Cota-Coronado A. Hernández-Pérez O. R. Lugo-Fabres P. H. Padilla-Camberos E. Díaz N. F. . (2020). iPSC for modeling neurodegenerative disorders. Regen. Ther. 15, 332–339. 10.1016/j.reth.2020.11.006 33426236 PMC7770414

[B200] van den Hurk M. Bardy C. (2019). Single-cell multimodal transcriptomics to study neuronal diversity in human stem cell-derived brain tissue and organoid models. J. Neurosci. Methods 325:108350. 10.1016/j.jneumeth.2019.108350 31310823

[B201] Venkataraman L. Fair S. R. McElroy C. A. Hester M. E. Fu H. (2020). Modeling neurodegenerative diseases with cerebral organoids and other three-dimensional culture systems: focus on Alzheimer's disease. Stem Cell Rev. Rep. 10, S10–S17. 10.1007/s12015-020-10068-9 33180261 PMC7658915

[B202] Vierbuchen T. Ostermeier A. Pang Z. P. Kokubu Y. Südhof T. C. Wernig M. (2010). Direct conversion of fibroblasts to functional neurons by defined factors. Nature 463, 1035–1041. 10.1038/nature08797 20107439 PMC2829121

[B203] Volpato V. Webber C. (2020). Addressing variability in iPSC-derived models of human disease: guidelines to promote reproducibility. Dis. Model. Mech. 13:dmm042317. 10.1242/dmm.042317 31953356 PMC6994963

[B204] Walus K. Beyer S. Willerth S. M. (2020). Three-dimensional bioprinting healthy and diseased models of the brain tissue using stem cells. Curr. Opin. Biomed. Eng. 14, 25–33. 10.1016/j.cobme.2020.03.002

[B205] Wang L. L. Serrano C. Zhong X. Ma S. Zou Y. Zhang C. L. (2021). Revisiting astrocyte to neuron conversion with lineage tracing *in vivo*. Cell 184, 5465–5481.e5416. 10.1016/j.cell.2021.09.005 34582787 PMC8526404

[B206] Wang M. Zhang L. Gage F. H. (2020). Modeling neuropsychiatric disorders using human induced pluripotent stem cells. Protein Cell 11, 45–59. 10.1007/s13238-019-0638-8 31134525 PMC6949328

[B207] Wang X. Hou Y. Ai X. Sun J. Xu B. Meng X. . (2020). Potential applications of microfluidics based blood brain barrier (BBB)-on-chips for *in vitro* drug development. Biomed. Pharmacother. 132:110822. 10.1016/j.biopha.2020.110822 33059264

[B208] Watanabe R. Buschauer R. Böhning J. Audagnotto M. Lasker K. Lu T. W. . (2020). The *in situ* structure of Parkinson's disease-linked LRRK2. Cell 182, 1508–1518.e1516. 10.1016/j.cell.2020.08.004 32783917 PMC7869717

[B209] Winbo A. Ramanan S. Eugster E. Jovinge S. Skinner J. R. Montgomery J. M. (2020). Functional coculture of sympathetic neurons and cardiomyocytes derived from human-induced pluripotent stem cells. Am. J. Physiol. Heart Circ. Physiol. 319, H927–H937. 10.1152/ajpheart.00546.2020 32822546

[B210] Workman M. J. Mahe M. M. Trisno S. Poling H. M. Watson C. L. Sundaram N. . (2017). Engineered human pluripotent-stem-cell-derived intestinal tissues with a functional enteric nervous system. Nat. Med. 23, 49–59. 10.1038/nm.4233 27869805 PMC5562951

[B211] Ximerakis M. Lipnick S. L. Innes B. T. Simmons S. K. Adiconis X. Dionne D. . (2019). Single-cell transcriptomic profiling of the aging mouse brain. Nat. Neurosci. 22, 1696–1708. 10.1038/s41593-019-0491-3 31551601

[B212] Xue Y. Fu Y. Zhao F. Gui G. Li Y. Rivero-Hinojosa S. . (2021). Frondoside A inhibits an MYC-driven medulloblastoma model derived from human-induced pluripotent stem cells. Mol. Cancer Ther. 20, 1199–1209. 10.1158/1535-7163.MCT-20-0603 33722850 PMC8172454

[B213] Yuva-Aydemir Y. Almeida S. Krishnan G. Gendron T. F. Gao F. B. (2019). Transcription elongation factor AFF2/FMR2 regulates expression of expanded GGGGCC repeat-containing C9ORF72 allele in ALS/FTD. Nat. Commun. 10:5466. 10.1038/s41467-019-13477-8 31784536 PMC6884579

[B214] Zahs K. R. Ashe K. H. (2010). 'Too much good news' - are Alzheimer mouse models trying to tell us how to prevent, not cure, Alzheimer's disease? Trends Neurosci. 33, 381–389. 10.1016/j.tins.2010.05.004 20542579

[B215] Zhang M. Vandana J. J. Lacko L. Chen S. (2020). Modeling cancer progression using human pluripotent stem cell-derived cells and organoids. Stem Cell Res. 49:102063. 10.1016/j.scr.2020.102063 33137568 PMC7849931

[B216] Zhang Y. Pak C. Han Y. Ahlenius H. Zhang Z. Chanda S. . (2013). Rapid single-step induction of functional neurons from human pluripotent stem cells. Neuron 78, 785–798. 10.1016/j.neuron.2013.05.029 23764284 PMC3751803

[B217] Zhao A. D. Qin H. Sun M. L. Ma K. Fu X. B. (2020). Efficient and rapid conversion of human astrocytes and ALS mouse model spinal cord astrocytes into motor neuron-like cells by defined small molecules. Mil. Med. Res. 7:42. 10.1186/s40779-020-00271-7 32892745 PMC7487818

[B218] Zhao J. Fu Y. Yamazaki Y. Ren Y. Davis M. D. Liu C. C. . (2020). APOE4 exacerbates synapse loss and neurodegeneration in Alzheimer's disease patient iPSC-derived cerebral organoids. Nat. Commun. 11:5540. 10.1038/s41467-020-19264-0 33139712 PMC7608683

[B219] Zheng X. Zhang L. Kuang Y. Venkataramani V. Jin F. Hein K. . (2021). Extracellular vesicles derived from neural progenitor cells–a preclinical evaluation for stroke treatment in mice. Transl. Stroke Res. 12, 185–203. 10.1007/s12975-020-00814-z 32361827 PMC7803677

[B220] Zhong S. Zhang S. Fan X. Wu Q. Yan L. Dong J. . (2018). A single-cell RNA-seq survey of the developmental landscape of the human prefrontal cortex. Nature 555, 524–528. 10.1038/nature25980 29539641

[B221] Zhu X. Zhou W. Jin H. Li T. (2018). Brn2 alone is sufficient to convert astrocytes into neural progenitors and neurons. Stem Cells Dev. 27, 736–744. 10.1089/scd.2017.0250 29635978

